# A signaling hub of insulin receptor, dystrophin glycoprotein complex and plakoglobin regulates muscle size

**DOI:** 10.1038/s41467-020-14895-9

**Published:** 2020-03-13

**Authors:** Yara Eid Mutlak, Dina Aweida, Alexandra Volodin, Bar Ayalon, Nitsan Dahan, Anna Parnis, Shenhav Cohen

**Affiliations:** Faculty of Biology, Technion Institute of Technology, Haifa, Israel

**Keywords:** Cell biology, Mechanisms of disease

## Abstract

Signaling through the insulin receptor governs central physiological functions related to cell growth and metabolism. Here we show by tandem native protein complex purification approach and super-resolution STED microscopy that insulin receptor activity requires association with the fundamental structural module in muscle, the dystrophin glycoprotein complex (DGC), and the desmosomal component plakoglobin (γ-catenin). The integrity of this high-molecular-mass assembly renders skeletal muscle susceptibility to insulin, because DGC-insulin receptor dissociation by plakoglobin downregulation reduces insulin signaling and causes atrophy. Furthermore, low insulin receptor activity in muscles from transgenic or fasted mice decreases plakoglobin-DGC-insulin receptor content on the plasma membrane, but not when plakoglobin is overexpressed. By masking β-dystroglycan LIR domains, plakoglobin prevents autophagic clearance of plakoglobin-DGC-insulin receptor co-assemblies and maintains their function. Our findings establish DGC as a signaling hub, and provide a possible mechanism for the insulin resistance in Duchenne Muscular Dystrophy, and for the cardiomyopathies seen with plakoglobin mutations.

## Abstract (web summary)

Insulin receptor signaling governs central physiological functions related to cell growth and metabolism. Here the authors use protein complex purification and super-resolution microscopy to show that insulin receptor activity requires association with dystrophin glycoprotein complex and plakoglobin.

## Introduction

The reciprocal dynamics between tissue architecture and function is maintained by the integrity of structural complexes and the transmission of growth signals from cell surface receptors. In the skeletal muscle, impaired structural integrity or loss of growth-promoting signals lead to reduced cell size and contractile capacity, and different myopathies in human^1,2^. Phosphoinositide 3-kinase-Akt (PI3K-Akt) signaling is transmitted via the insulin receptor, and reduced activity of this pathway causes muscle atrophy, and largely contributes to the development of insulin resistance in type 2 diabetes^3–6^. A similar reduction in insulin sensitivity^7,8^ can be seen in some Duchenne muscular dystrophy (DMD) patients, a disease caused by dissociation of dystrophin glycoprotein complex (DGC)^9,10^, although the precise mechanism is unknown. Specifically, it is unclear how the integrity of the pivotal structural unit of the skeletal muscle, the DGC, affects insulin-PI3K-Akt signaling. Here, we demonstrate that the DGC is physically and functionally linked to the insulin receptor on the plasma membrane.

The DGC plays a structural role by anchoring the cytoskeleton to the extracellular matrix (ECM) to provide mechanical support and protect the sarcolemma against stress imposed during muscle contraction^11^. It is composed of dystrophin, dystroglycans, sarcoglycans, sarcospan, dystrobrevins, and syntrophin^11,12^, and loss of dystrophin causes DMD. In skeletal muscle, the DGC is confined to subsarcolemmal structures called “costameres,” which reside at the sarcolemma in register with the subjacent sarcomeric Z-bands, just where desmin filaments abut the plasma membrane^13^. In addition, DGC seems to regulate signaling pathways that control cell growth and differentiation^12,14–16^, and DGC dissociation in muscles from DMD patients sometimes correlates with the development of insulin resistance^7,8^. Furthermore, reduced DGC integrity in tumor-bearing mice induces the expression of atrophy-related genes and promotes muscle wasting^14^. The present studies uncover a signaling hub of DGC and insulin receptor, which resides in costameres and whose integrity and function require the desmosomal component plakoglobin.

Plakoglobin is a component of desmosome adhesion complexes that are prominent in tissues that must withstand mechanical stress, especially cardiomyocytes and epithelia^17,18^. In epithelia, plakoglobin regulates signaling pathways (e.g., by Wnt) that control cell motility^19^, growth, and differentiation^20^. We have recently shown that plakoglobin is of prime importance in regulating skeletal muscle size because it binds to the insulin receptor and enhances the activity of the PI3K-Akt pathway^21^. Activation of this pathway by insulin-like growth factor-1 (IGF-I) or insulin promotes glucose uptake and overall protein synthesis, and inhibits protein degradation^2,22,23^. In untreated diabetes, sepsis, and cancer cachexia^24^, impaired signaling through this pathway causes severe muscle wasting, which can be inhibited by the activation of PI3K-Akt-FoxO signaling^25^. Because changes in plakoglobin levels alone influence PI3K-Akt-FoxO pathway^21^, perturbation of its function may contribute to insulin resistance in disease. We show here that plakoglobin is important for the maintenance of muscle size by stabilizing DGC and mediating its association with and activation of the insulin receptor.

## Results

### Isolation of plakoglobin complexes from the skeletal muscle

**Fig. 1:**
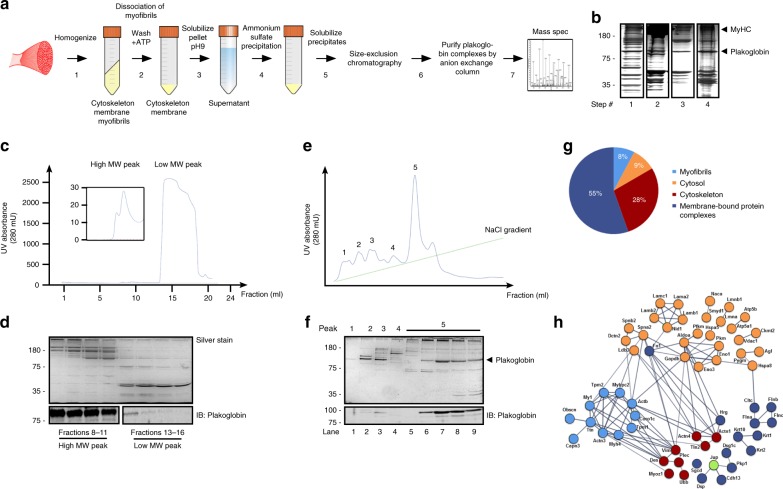
Isolation of plakoglobin-containing protein complexes from mouse skeletal muscle. **a** Scheme of the native protein complex purification and proteomic approach. **b** Input from steps 1–4 in **a** was analyzed by SDS-PAGE and silver staining (a representative of three independent analyses). Black lines indicate the removal of intervening lanes for clarity and presentation purposes. **c** Size-exclusion chromatography of solubilized ammonium sulfate precipitates from step 5 in **a** yielded two distinct high and low MW protein peaks. **d** SDS-PAGE of fractions from the high (fractions #8–11) and low (fractions #13–16) MW protein peaks in **c**, followed by silver staining (top panel) or immunoblotting with plakoglobin antibody (lower panel) (a representative of three independent analyses). Black lines indicate the removal of intervening lanes for clarity and presentation purposes. **e** Isolation of plakoglobin-containing protein complexes by anion exchange chromatography. Fractions #8–11 from high MW protein peak in **d** were pooled and applied to a Resource Q column, and protein complexes were isolated by a 0–500 mM NaCl gradient. **f** Analysis of eluted peak fractions presented in **e** by SDS-PAGE and silver staining or immunoblotting using plakoglobin antibody. To identify the components that co-eluted with plakoglobin from the Resource Q column, lane 7 in protein peak #5 was subjected to mass spectrometry analysis. **g** Distribution of proteins that co-eluted with plakoglobin from the anion exchange column (protein peak #5, lane 7 in **e**) into DAVID-derived categories, and percentages of components assigned to each category. **h** Interaction networks for components identified in **g** using the STRING database. Proteins are grouped by cellular distribution. Colors of individual proteins correspond to the distribution presented in **g**. Active interactions (textmining, experiments, databases) are based on published data, using interaction score of high confidence (0.7).

To understand plakoglobinʼs role in the maintenance of normal muscle size, we isolated the complexes that it forms in skeletal muscle. Using a modified protocol^26^, we washed pellets from insoluble fractions of lower limb mouse muscles (the 10,000 × *g* pellet) with high ATP buffer to dissociate the myofibrils, and high pH buffer (pH 9 at 37 °C) to solubilize cytoskeletal and membrane-bound protein complexes (Fig. 1a, b). Ammonium sulfate precipitates were then analyzed by gel filtration column (Superdex 200 10/300), and showed two distinct protein peaks containing high and low molecular weight (MW) proteins (Fig. 1c, d). A similar two-peak elution profile has been obtained from rat skeletal muscles (Supplementary Fig. 1). Plakoglobin was predominantly enriched in the high MW protein peak, and thus seems to be a component of multiprotein assemblies (Fig. 1d). In fact, analysis of the high MW protein peak by a Resource Q anion exchange column indicated that plakoglobin was eluted at a high yield together with several high MW polypeptides as a major monodisperse sharp peak at 250 mM NaCl (peak #5) (Fig. 1e, f). Mass spectrometry analysis (Fig. 1f, lane 7) identified mainly cytoskeletal (28%) and membrane-bound (55%) proteins, including components comprising costameres, such as the DGC component δ-sarcoglycan, the basal lamina glycoprotein laminin, which binds to the DGC, and the dystrophin-associated protein spectrin^27^ (Fig. 1g, h and Supplementary Table 1^28^). Furthermore, the major intermediate filament protein in the muscle, desmin, was identified, as well as the associated proteins desmoplakin^29^, the desmin cross-linking protein plectin, and α-actinin, a component localized to the Z-bands where desmin filaments are aligned (Supplementary Table 1).

**Fig. 2:**
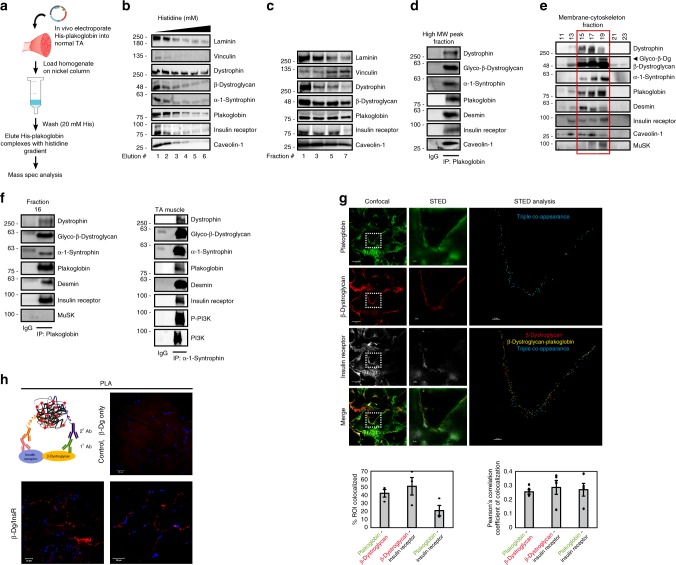
Plakoglobin binds DGC components and insulin receptors at costameres on the plasma membrane. **a** Affinity-based purification of plakoglobin-containing complex. 6His-tagged plakoglobin encoding plasmid was electroporated into muscle, and 6His-plakoglobin-bound proteins were isolated with Nickel beads and identified by mass spectrometry. **b** 6His-plakoglobin-bound proteins were eluted from Nickel column with histidine gradient (50–250 mM) and analyzed by immunoblotting. Fraction #2 was subjected to mass spectrometry. *n* = one experiment, data were compared to Fig. 1g, h. **c** Analysis of high MW protein fractions (as in Fig. 1c) by immunoblotting. *n* = two independent experiments. **d** Plakoglobin immunoprecipitation from the high MW protein peak. Bound proteins were detected by immunoblotting. **e** Plakoglobin, DGC components (including glycosylated-β-dystroglycan), the insulin receptor, caveolin-1, desmin, and MuSK sediment to the same glycerol gradient fractions (marked by a red rectangle). Membrane-cytoskeleton fraction from mouse muscle was analyzed by 10–40% glycerol gradient and immunoblotting. *n* = two independent experiments. **f** In normal muscle, plakoglobin, DGC components, the insulin receptor, and desmin interact. Left: Proteins co-purified with anti-plakoglobin from fraction #16 in **e**, and detected by immunoblotting. MuSK did not bind plakoglobin, although it sedimented to the same fractions. Right: Reciprocal immunoprecipitation with α-1-syntrophin antibody from membrane fractions isolated from normal muscle. Two experiments were performed: One with plakoglobin antibody and one with α-1-syntrophin antibody. **g** Plakoglobin, β-dystroglycan, and the insulin receptor potentially colocalize at costameres on the skeletal muscle membrane. STED: Stimulated emission depletion microscopy. Confocal (bar, 5 μm) and STED (bar, 2 μm) images of muscle cross-sections stained with the indicated antibodies. *n* = three independent experiments. STED analysis was performed using the spots module of the Imaris software for the three proteins. The double and triple co-occurrence spots are presented in yellow (plakoglobin-β-dystroglycan) and blue (plakoglobin-β-dystroglycan-insulin receptor). Right: Percent colocalization of indicated proteins in region of interest (the region of co-occurrence, confocal images), and the corresponding Pearson’s correlation coefficients of colocalization. *n* = 3, and two independent experiments. Data are represented as mean ± SEM. **h** Proximity ligation assay (PLA) was performed on TA cross-sections with β-dystroglycan and insulin receptor antibodies or β-dystroglycan antibody alone. Red fluorescent dots indicate areas of β-dystroglycan-insulin receptor association.

To confirm these findings by an independent approach, and identify additional proteins that bind plakoglobin, we transfected mouse tibialis anterior (TA) muscle with a plasmid encoding 6His-plakoglobin and isolated bound proteins with a Nickel column (Fig. 2a). Mass spectrometry analysis identified most of the aforementioned components as plakoglobin-bound proteins, and additional DGC core components, including dystrophin, α-sarcoglycan, β-sarcoglycan, γ-sarcoglycan, α-syntrophin, β-syntrophin, and β-dystroglycan, the costamere marker in the skeletal muscle, vinculin, where DGC is normally located^13,15^, and the specialized lipid membrane domain caveolae markers, caveolin-1 and -3, which interact with DGC in striated muscle and link the cytoskeleton to the ECM^30,31^ (Supplementary Table 2). Other plasma membrane-bound proteins included insulin signaling components such as the p85 regulatory subunit of PI3K, which we previously showed can bind plakoglobin^21^, the insulin receptor substrate 1, which specifically binds to the insulin receptor and activates PI3K-Akt signaling^32^, two catalytic subunits of PI3K, type 3 and gamma, that bind p85-PI3K to form the PI3K complex, the insulin-like growth factor 2 receptor, and the insulin-like growth factor-binding protein (Supplementary Table 2).

### DGC, insulin receptors, and plakoglobin physically interact

To learn whether DGC, insulin receptors, and plakoglobin form one intact co-assembly, we repeated 6His-plakoglobin isolation from transfected TA muscles using a Nickel column, and eluted the bound proteins with a histidine gradient. DGC components, vinculin, caveolin-1, the insulin receptor, and laminin, were effectively co-purified with 6His-plakoglobin (Fig. 2b). These proteins physically interact in vivo at membrane caveolae because DGC components, insulin receptors, and caveolin-1 co-precipitated together with plakoglobin from the high MW protein peak (Fig. 2c, d). Moreover, plakoglobin, DGC components, desmin, insulin receptor, and p85-PI3K sedimented to the same glycerol gradient fractions (Fig. 2e), and formed a native multimeric assembly because they co-precipitated together with plakoglobin from these fractions, and also reciprocally with α-syntrophin (Fig. 2f). β-Dystroglycan appeared in its glycosylated form (Fig. 2e), which is essential for DGC stability^11^. Also, the presence of desmin in plakoglobin-containing precipitates is consistent with the structural role of plakoglobin in linking the desmin cytoskeleton through membrane structures (e.g., DGC) to the ECM^33,34^. These interactions were specific because tyrosine kinase receptor, MuSK, which was also present in these fractions, was not precipitated with plakoglobin antibody (Fig. 2e, f and Supplementary Fig. 2). Similar interactions were demonstrated by coimmunoprecipitation in the heart and liver (Supplementary Fig. 2), indicating that these interactions are not unique to the skeletal muscle, and plakoglobin probably binds, and thus might regulate, these structural and signal transmitting modules in many (perhaps all) tissues.

Immunofluorescence staining of muscle cross-sections and analysis by confocal and super-resolution stimulated emission depletion (STED) microscopy confirmed that plakoglobin potentially colocalize with β-dystroglycan, insulin receptors (Fig. 2g), dystrophin and vinculin (Supplementary Fig. 3a) on the fiber membrane. Sub-diffraction co-occurrence analysis of STED images and three-dimensional analysis of confocal images confirmed a distinct heteromultimeric assembly of DGC-insulin receptor-plakoglobin with vinculin (Fig. 2g and Supplementary Fig. 3a, b). These interactions were corroborated by a proximity ligation assay (PLA), which allows in situ detection of protein–protein interactions. Muscle cross-sections were incubated with primary antibodies against insulin receptor and β-dystroglycan and secondary antibodies linked to complementary DNA probes. Because target proteins are 20–100 nm apart, the probes could form a closed DNA circle, which served as a template for rolling circle amplification reaction, and subsequent incorporation of fluorescently labeled nucleotides. Consequently, the red fluorescent spots along the plane of the fiber membrane indicate areas where the insulin receptor and β-dystroglycan interact (Fig. 2h). These red spots were not formed in muscle sections incubated with β-dystroglycan antibody alone (Fig. 2h). Interestingly, these discrete regions are located in close proximity to the nucleus, most likely to efficiently translate signals from the plasma membrane (e.g., PI3K-Akt signaling) to transcriptional response (Supplementary Fig. 3c). Together, these findings indicate that in the skeletal muscle, and probably other tissues, plakoglobin co-assembles with DGC and the insulin receptor on the plasma membrane, and may be important for bridging and stabilizing these structures.

### Plakoglobin links insulin receptor activity to DGC integrity

**Fig. 3:**
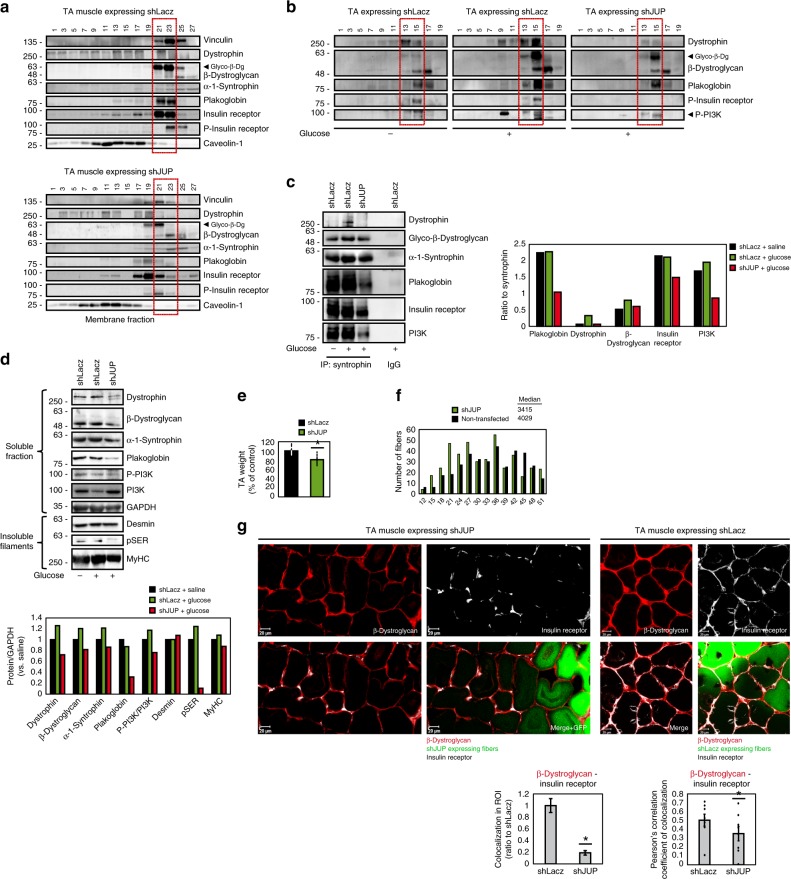

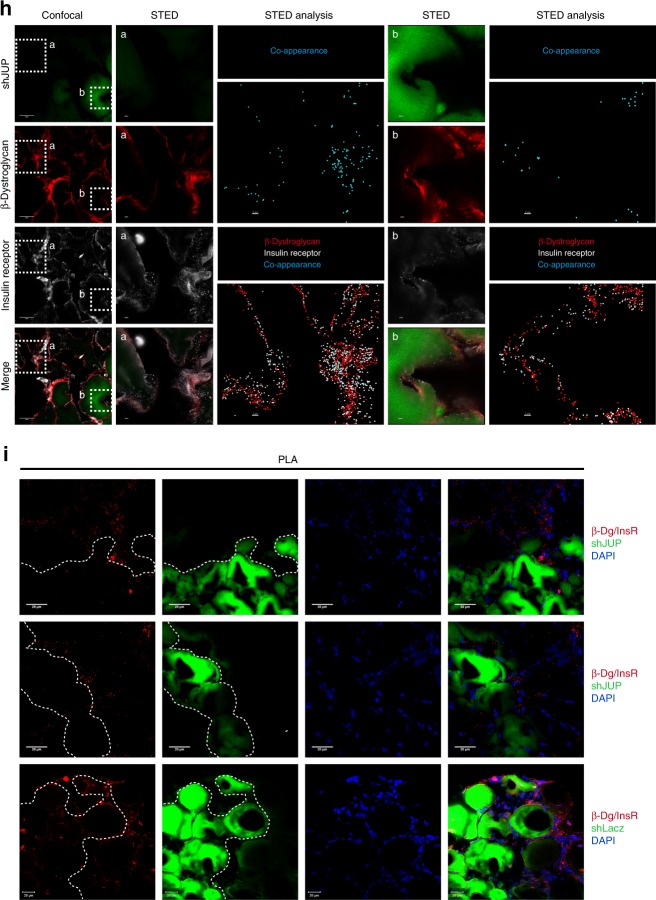
Plakoglobin downregulation reduces DGC-insulin receptor association and insulin signaling, and causes atrophy. Contralateral muscles from fasted mice were electroporated with plakoglobin shRNA (shJUP) or control shRNA (shLacz). Red rectangle marks fractions containing glycosylated-β-dystroglycan in shLacz-expressing muscles. STED: Stimulated emission depletion microscopy. **a** Muscle membrane extracts were analyzed by glycerol gradients and immunobloting. *n* = three independent experiments. **b** Mice were injected with saline or glucose (1 mg/g body weight) and muscle membrane extracts were analyzed by glycerol gradient and immunoblotting. *n* = three independent experiments. **c** Syntrophin immunoprecipitation from muscle membrane extracts from mice injected with saline or glucose (1 mg/g body weight). Precipitates were analyzed by immunoblotting. Right: Densitometric measurements of presented blots. Graph depicts ratio of each protein to syntrophin. *n* = two independent experiments. **d** Soluble fractions of transfected muscles from mice injected with saline or glucose were analyzed by immunobloting. *n* = two independent experiments. **e** Mean weights of muscles expressing shJUP are plotted as the percentage of shLacz muscles. Data are represented as mean ± SEM. *n* = 14 mice; *P < 0.00005 by one-tailed *t* test. *n* = two independent experiments. **f** Cross-sectional areas of 521 fibers expressing shJUP (also express GFP, green bars) vs. 521 non-transfected fibers (black bars). *n* = 4 mice. **g** Top: Representative confocal images of transfected muscles cross-sections (also express GFP), stained with anti-insulin receptor and anti-β-dystroglycan. Bar, 20 μm. *n* = two independent experiments. Bottom: Colocalization of β-dystroglycan and insulin receptor in region of interest (the region of co-occurrence, data are presented as ratio to shLacz), and the corresponding Pearson’s correlation coefficients of colocalization. *n* = 8, two independent experiments. *P < 0.05 vs. shLacz, by one-tailed *t* test. Data are represented as mean ± SEM. **h** Confocal (bar, 5 μm) and STED (bar, 2 μm) images of cross-sections of transfected muscles stained with the indicated antibodies. *n* = two independent experiments. STED analysis was performed using the spots module of the Imaris software for β-dystroglycan and insulin receptor (white and red spheres, respectively). β-Dystroglycan-insulin receptor co-occurrence is in blue. **i** Proximity ligation assay (PLA) was performed on cross-sections of transfected muscles stained with β-dystroglycan and insulin receptor antibodies. Red fluorescent dots indicate areas of β-dystroglycan-insulin receptor association.

To test whether plakoglobin enhances the structural integrity and functional properties of DGC and insulin receptor, we downregulated plakoglobin in normal muscle by electroporation of short hairpin RNA (shRNA) (shJUP)^21^, and analyzed the effects on DGC stability and insulin-PI3K-Akt signaling. Glycerol gradient fractionation of purified membranes from transfected muscles showed that most proteins sedimented to the same fractions, but not when plakoglobin was downregulated (Fig. 3a). Plakoglobin loss led to decreased insulin receptor phosphorylation, and caused a marked reduction in glycosylated β-dystroglycan on the membrane, suggesting that DGC was destabilized^11^. Furthermore, the content of vinculin, dystrophin, and the insulin receptor on the membrane was also reduced and their distribution was altered in shJUP-expressing muscles, strongly suggesting that the integrity of plakoglobin-containing complex was compromised (Fig. 3a). This reduction in the membrane content of DGC components and insulin receptor is selective because in the muscles expressing shJUP, the protein levels of caveolin-1 and the unmodified form of β-dystroglycan were similar to shLacz controls (Fig. 3a).

Consistently, glucose injection to mice to induce insulin secretion activated insulin signaling (compared with mice injected with saline) (Fig. 3b), and enhanced DGC-insulin receptor association (Fig. 3c), but not in the muscles expressing shJUP, where insulin receptor and PI3K phosphorylation was markedly reduced, and much less of these proteins were associated in one intact complex (Fig. 3a–c). Similar effects were obtained in muscles expressing shJUP or shLacz from mice injected with saline (Supplementary Fig. 4a). Interestingly, the soluble pools (the fraction soluble at 6000 × *g*) of DGC subunits (i.e., dystrophin, β-dystroglycan, α-syntrophin) were smaller when plakoglobin was downregulated (Fig. 3d) and insulin signaling fell (Fig. 3a, b, d), possibly due to their degradation. Loss of plakoglobin and the resulting reduction in DGC integrity also enhanced depolymerization of the desmin cytoskeleton because its phosphorylated species decreased in the insoluble fraction (Fig. 3d)^35–37^. In fact, these effects by shJUP must underestimate the actual effects of plakoglobin downregulation because only about 70% of the fibers were transfected (Supplementary Fig. 4b).

As expected from the fall in PI3K-Akt signaling, in the muscles expressing shJUP atrophy became evident because the mean TA weights was lower by 20% than control (Fig. 3e), and the mean cross-sectional area of 500 fibers expressing shJUP was smaller than 500 non-transfected ones (Fig. 3f)^21^. Finally, immunofluorescence staining using confocal (Fig. 3g) and STED (Fig. 3h) microscopy, as well as PLA (Fig. 3i), confirmed insulin receptor-β-dystroglycan potential colocalization and association on the sarcolemma of non-transfected or shLacz-expressing fibers, which was markedly reduced in fibers expressing shJUP (Fig. 3g–i). Therefore, plakoglobin is required for DGC stability, which appears to be linked to normal signaling through the insulin receptor.

### Inhibition of insulin receptor function reduces association with DGC

**Fig. 4:**
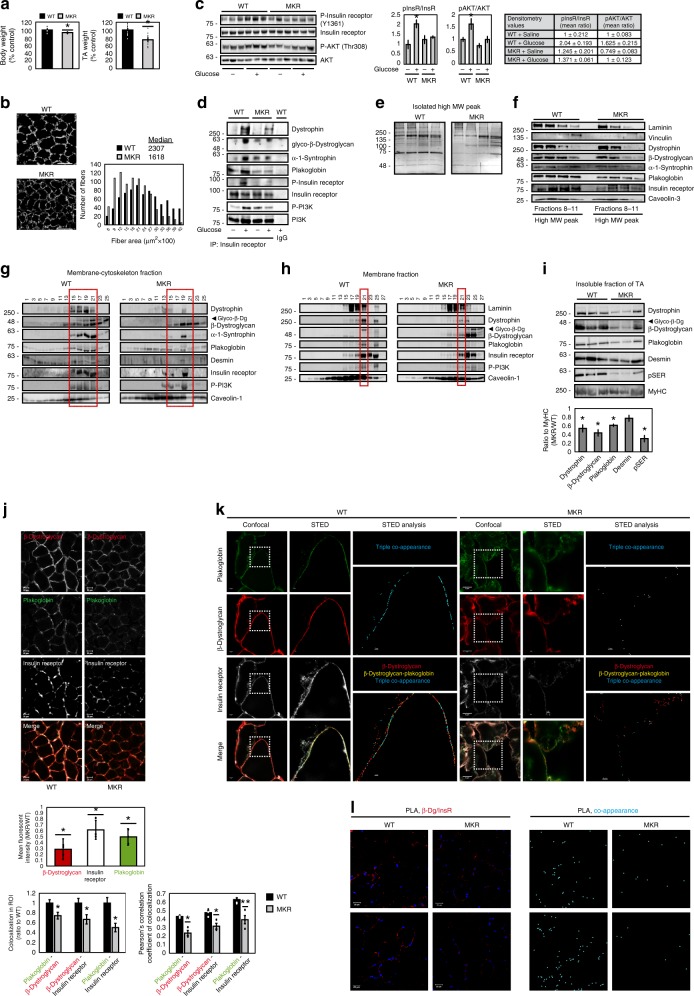
In MKR mouse muscles insulin receptor-DGC association is reduced. **a** Mean body (*n* = 5) and TA (*n* = 12) weights of MKR mice at 8 weeks old are presented as percentage of WT. *P < 0.05, by one-tailed *t* test. Data are mean ± SEM. *n* = two independent experiments. **b** Left: Laminin staining (white). Bar, 50 μm. Right: Cross-sectional areas of 834 fibers from WT (black bars) and MKR (gray bars) mice. *n* = 3 mice. **c** Left: Soluble fractions of mouse muscles injected with saline or glucose were analyzed by immunoblotting. *n* = two independent experiments. Right: Densitometric measurement of presented blots. **d** Insulin receptor immunoprecipitation from muscle membranes of mice injected with saline or glucose. *n* = two independent experiments. **e**, **f** High MW protein peak fractions isolated from mouse muscles were analyzed by silver staining and immunoblotting. *n* = three independent experiments. **g** Membrane-cytoskeleton fractions from mouse muscles were analyzed by glycerol gradients. Red rectangle marks fractions containing glycosylated-β-dystroglycan in WT. *n* = two independent experiments. **h** Isolated mouse muscle membranes were analyzed by glycerol gradients. *n* = two independent experiments. Red rectangle marks fractions containing glycosylated-β-dystroglycan in WT. **i** Top: Muscle insoluble fractions were analyzed by immunoblotting. *n* = two independent experiments. Bottom: Densitometric measurement of presented blots. **j** Top: Muscle cross-sections were stained with plakoglobin, β-dystroglycan, and insulin receptor antibodies. Bar, 20 μm. *n* = three independent experiments. Middle: Fluorescent intensity was quantified using the Imaris software. Bottom: Colocalization of indicated proteins in region of interest (region of co-occurrence, data are ratio to WT), and the corresponding Pearson’s correlation coefficients of colocalization. *n* = 6, and two independent experiments. *P < 0.05 and **P < 0.005 vs. WT, by one-tailed *t* test. Data are mean ± SEM. **k** STED: Stimulated emission depletion microscopy. Confocal (bar, 5 μm) and STED (bar, 2 μm) images of muscles cross-sections stained with indicated antibodies. *n* = two independent experiments. STED analysis was performed using the spots module of Imaris software for the three proteins. The double and triple co-occurrence spots are presented in yellow (plakoglobin-β-dystroglycan) and blue (plakoglobin-β-dystroglycan-insulin receptor). **l** Proximity ligation assay (PLA) was performed on muscle cross-sections stained with β-dystroglycan and insulin receptor antibodies. Red fluorescent dots (left) and blue dot analysis (right) indicate areas of β-dystroglycan-insulin receptor association.

Altogether, these observations suggested that DGC integrity is linked to insulin receptor activity via plakoglobin. To test this idea and investigate whether the membrane content of DGC is influenced by the fall in PI3K-Akt signaling, we used transgenic mouse  (MKR) model for type 2 diabetes, which express a kinase-inactive form of IGF-1 receptor (IGF-1R) specifically in skeletal muscle. This IGF-1R mutant dimerizes and inhibits IGF-1R and insulin receptor activities ultimately leading to skeletal muscle-specific inhibition of PI3K-Akt signaling, and insulin resistance^4,38^. Consistent with prior studies^38^, at 8 weeks of age, the mean body weights of MKR mice were 7% lower, the mean wet weights of their TA muscles were 25% lower, and the cross-sectional area of their muscle fibers was smaller than age-matched control mice (Fig. 4a, b). This reduction in muscle size probably resulted from the fall in PI3K-Akt signaling, which could not be activated even when glucose was injected to stimulate insulin secretion (Fig. 4c, d)^38^. Accordingly, glucose injection induced association of PI3K, plakoglobin, and DGC components with the insulin receptor in muscles from wild-type (WT) mice, but failed to do so in muscles from MKR mice (Fig. 4c, d). Thus, impaired insulin receptor function reduces its association with DGC.

To learn how this fall in PI3K-Akt signaling influences DGC integrity, we analyzed MKR mouse muscles by gel filtration chromatography (as in Fig. 1). The high MW protein peak fractions from MKR mouse muscles contained lower amounts of plakoglobin-DGC-insulin receptors co-assembly than WT littermates (Fig. 4e, f). These proteins were probably degraded because they did not accumulate in the soluble fraction (Supplementary Fig. 5a). Although caveolin-3 levels in MKR muscles were similar to WT, the amounts of laminin and vinculin were reduced, suggesting that in MKR mouse muscles the structural link between the cytoskeleton and laminin at the ECM, via vinculin and DGC, is perturbed (Fig. 4f).

These findings were corroborated biochemically by glycerol gradient fractionations of membrane-cytoskeleton preparations (Fig. 4g) and membrane extracts (Fig. 4h), which indicated decreased amounts of DGC-insulin receptor co-assemblies and glycosylated β-dystroglycan in MKR mice muscles than in control, most likely due to the fall in the membrane content of plakoglobin (Figs. 3 and 4g, h), which is sufficient to cause DGC-insulin receptor dissociation (Fig. 3). In these muscles, phosphorylated desmin filaments were lost (Fig. 4i), as occurs in atrophying muscles from mouse and human^35–37,39^, which did not result from reduced gene expression because the mRNA levels of desmin, DGC components, and plakoglobin were similar in muscles from WT and MKR mice (Supplementary Fig. 5b). The insoluble fraction (6000 × *g* pellet) from MKR mouse muscles, which constitute the structural linkage of the myofibrils through desmin to membrane structures (e.g., DGC), contained fewer components of plakoglobin-containing heteromultimers, and their loss exceeded the reduction in myofibrillar myosin heavy chain (Fig. 4i). Finally, an overall reduction in the content of plakoglobin-DGC-insulin receptor clusters on the muscle membrane of MKR than WT mice was demonstrated by confocal, STED and PLA analyses (Fig. 4j–l), where plakoglobin seemed to accumulate inside the muscle fiber (Fig. 4k and Supplementary Fig. 5c, d). The mean fluorescent intensity of these components was reduced differentially because the average fluorescent intensity of wheat germ agglutinin (WGA) in the same muscles was not lower than in WT (Supplementary Fig. 5e). In addition, sub-diffraction co-occurrence analysis of STED images indicated a marked decrease in the number of protein clusters (Fig. 4k), and PLA analysis using insulin receptor and β-dystroglycan antibodies showed a decrease in insulin receptor-β-dystroglycan association (Fig. 4l) in muscles from MKR mice compared with WT. This loss of plakoglobin, or reduced function^21^, may be an early event leading to impaired insulin signaling and reduced DGC integrity in atrophy and disease.

### Plakoglobin prevents DGC-insulin receptor loss in disease

**Fig. 5:**
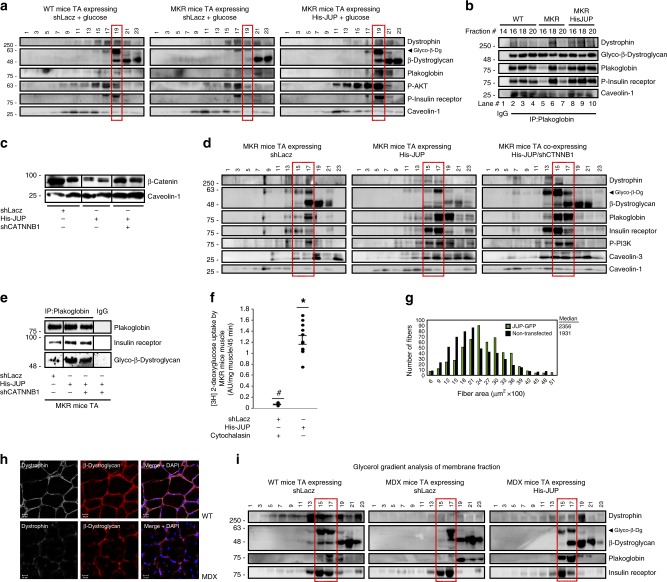
Overexpression of plakoglobin alone enhances DGC-insulin receptor association and insulin signaling in muscles from MKR or *mdx* mice. Muscles from WT or diabetic MKR mice were transfected with 6His-plakoglobin (His-JUP) or control (shLacz) plasmids (contralateral limbs). **a** Mice were injected with glucose (1 mg/g body weight) and membrane extracts from transfected muscles were analyzed by glycerol gradient and immunoblotting. *n* = three independent experiments. **b** Plakoglobin immunoprecipitation from glycerol gradient fractions presented in **a**. *n* = two independent experiments. **c**–**e** Membrane extracts from muscles expressing shLacz or β-catenin shRNA (shCTNNB1), with or without the co-electroporation of His-plakoglobin, were analyzed by immunoblotting (**c**), glycerol gradient fractionation (**d**), and immunoprecipitation (**e**). Black lines indicate the removal of intervening lanes for clarity and presentation purposes. *n* = two independent experiments. **f** [^3^H]-2-deoxyglucose uptake by muscles expressing shLacz in the presence of cytochalasin B (a competitive inhibitor of regulated glucose transport into cells) or His-JUP is plotted as ratio to shLacz control, which was not treated with cytochalasin B. Data are presented as AU/mg muscle/45 min. Data are mean ± SEM, *n* = 11 mice for His-JUP and shLacz, *n* = 4 mice for shLacz treated with cytochalasin B, *P = 0.01 and ^#^*P* = 0 vs. shLacz in the absence of cytochalasin B, by one-tailed *t* test. *n* = two independent experiments. **g** Overexpression of plakoglobin in muscles from MKR mice attenuates the reduction in fiber size. Measurement of cross-sectional area of 530 fibers expressing His-JUP (also express GFP; green bars) vs. 530 non-transfected fibers (black bars) in the same muscle. Data are acquired from seven mice. **h** Cross-sections of muscles from WT or *mdx* mice were immunostained with antibodies against dystrophin and β-dystroglycan (bar, 20 μm). *n* = two independent experiments from two different mice (representative confocal images are shown). **i** Membrane extracts from muscles expressing shLacz or His-JUP from WT and *mdx* mice and analyzed by glycerol gradient fractionation and immunoblotting. Transfected *mdx* mice muscles are from contralateral limbs. *n* = two independent experiments.

These observations suggested that increasing the level of plakoglobin in MKR mice muscle should enhance DGC-insulin receptor association, and accentuate insulin signaling. Accordingly, plakoglobin-DGC-insulin receptor heteromultimers were compromised and PI3K-Akt signaling fell in muscles from MKR mice compared with WT, but not when 6His-plakoglobin was overexpressed (Fig. 5a, b). Interestingly, in MKR mouse muscles, glycosylated β-dystroglycan remained bound to plakoglobin (Fig. 5b, lanes 5–7), suggesting an altered distribution of β-dystroglycan on the membrane when its association with the insulin receptor and other DGC components is low. This beneficial effect of plakoglobin overexpression did not require the function of its close homolog, β-catenin, because in the MKR mouse muscles expressing 6His-tagged plakoglobin, the simultaneous downregulation of β-catenin with shRNA (shCTNNB1) (Fig. 5c) did not perturb plakoglobin-DGC-insulin receptor association (Fig. 5d, e). Plakoglobin overexpression also enhanced glucose uptake by MKR mouse muscles (Fig. 5f) (cytochalasin B is a cell-permeable mycotoxin that competitively inhibits glucose transport into cells, and was used here to evaluate specifically regulated glucose uptake^40^), and markedly attenuated muscle fiber atrophy (Fig. 5g).

In light of these findings in MKR mouse muscles, we investigated whether loss of DGC integrity in muscles from *mdx* mice influences insulin signaling, and the role of plakoglobin. In muscles from *mdx* mice, dystrophin was absent and β-dystroglycan was reduced compared with WT (Fig. 5h). Consistent with our findings above in MKR mouse muscles, the membrane content of glycosylated β-dystroglycan-plakoglobin-insulin receptor in *mdx* mice was lower than WT, but not when plakoglobin was overexpressed (Fig. 5i). Thus, plakoglobin regulates muscle size by linking insulin receptor activity to DGC integrity.

### DGC-insulin receptor-plakoglobin interaction domain mapping

Co-assembly of DGC, the insulin receptor, and plakoglobin was demonstrated by mapping the sites on plakoglobin that constitute their interaction. Plakoglobin is composed of an N-terminal head domain, a C-terminal tail domain, and a central conserved domain containing 12 armadillo repeats^41^. Mutations within the N-terminal domain reduce plakoglobin membrane localization and cause cardiomyopathy in human^42^, and deletion of the C-terminal domain causes Naxos disease^43,44^. To identify the critical domains in plakoglobin that are required for its heterodimerization with DGC and the insulin receptor, we generated 6His-V5-tagged truncation mutants of plakoglobin lacking the N-terminal (ΔN), the C-terminal (ΔC), or either end (ΔNC) domains, while leaving the central armadillo domain intact (Supplementary Fig. 6a). Overexpression of ΔN-plakoglobin in normal muscle for 6 days resulted in decreased insulin receptor phosphorylation compared with muscles expressing full-length (FL) plakoglobin (Supplementary Fig. 6b), strongly suggesting that the plakoglobin N terminus is required for interaction with and activation of the insulin receptor. Consistently, affinity purification of plakoglobin truncation mutants from transfected muscles using Nickel column and histidine gradient (Supplementary Fig. 6c), and subsequent densitometric measurement of protein ratios indicated that deletion of plakoglobin N terminus (ΔN) reduced its association only with the insulin receptor, but not with β-dystroglycan or dystrophin, compared with FL-plakoglobin (Supplementary Fig. 6d). By contrast, plakoglobin-ΔC, which promotes formation of large abnormal membrane structures in Naxos disease^43,44^, showed increased association with both the insulin receptor and β-dystroglycan, and compensated for the loss of plakoglobin N terminus in plakoglobin-ΔNC, which remained bound to the insulin receptor (Supplementary Fig. 6d). These interactions were specific because none of these proteins bound the Nickel column in control muscles expressing shLacz (Supplementary Fig. 6c). Therefore, insulin receptor interacts with plakoglobin N terminus, and β-dystroglycan binds to sites adjacent to this region, which seem to be regulated by plakoglobin C-terminal tail. Interestingly, dystrophin bound similarly to FL-plakoglobin and all truncation mutants (Supplementary Fig. 6d), suggesting that its binding sites are located within plakoglobin central armadillo repeats domain. Plakoglobin may serve a scaffold linking DGC and insulin receptors physically and functionally to maintain muscle mass.

### Dynamics of plakoglobin-DGC-insulin receptor association

**Fig. 6:**
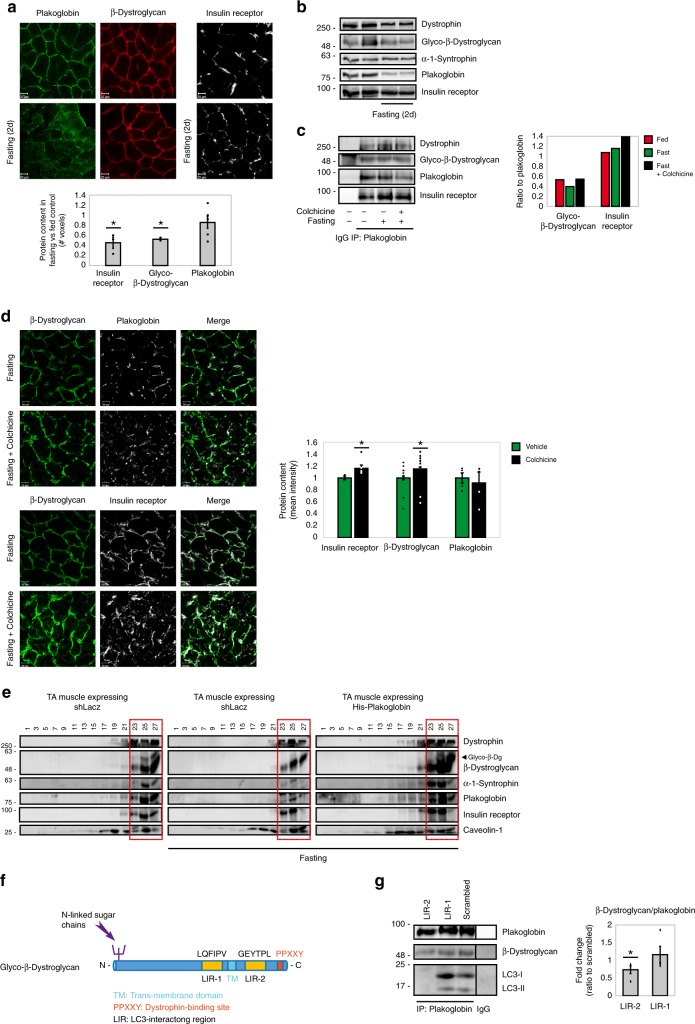

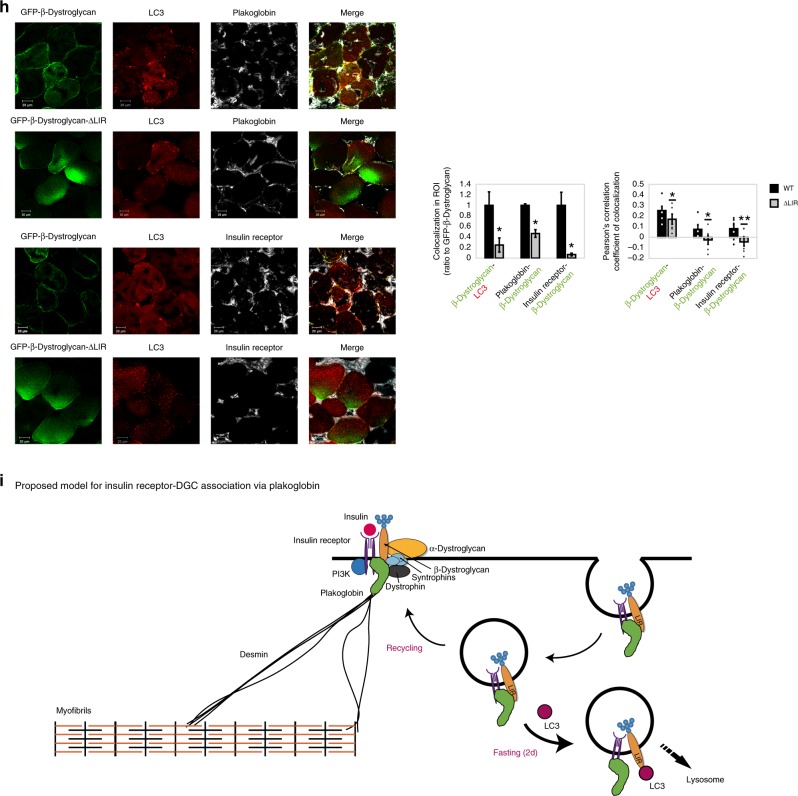
Plakoglobin-DGC-insulin receptor association is regulated by autophagy. Muscles from fed and fasted (2 days) mice are analyzed. In fasting, contralateral limbs were transfected. Data are mean ± SEM. *P* values by one-tailed *t* test. Fluorescence intensity was obtained using the Imaris software. **a** Top: Muscle cross-sections stained with indicated antibodies. Bar, 20 μm. *n* = four independent experiments. Bottom: Fluorescence intensity. *n* = 4. *P < 0.05 vs. fed. **b** Equal membrane extracts from muscles were analyzed by immunoblotting. *n* = two independent experiments. **c** Left: Plakoglobin immunoprecipitation from muscle membranes from mice treated with colchicine or vehicle was analyzed by immunoblotting. *n* = two independent experiments. Right: Densitometric measurement of presented blots. **d** Left: Muscle cross-sections from fasted mice treated with colchicine or vehicle were stained with indicated antibodies. Bar, 20 μm. *n* = three independent experiments. Right: Fluorescence intensity. *n* = 3. *P < 0.05 vs. vehicle injection. **e** Membranes purified from muscles expressing shLacz or 6His-plakoglobin were analyzed by glycerol gradients and immunoblot. Red rectangle marks fractions containing glycosylated-β-dystroglycan in shLacz-expressing muscles. *n* = four independent experiments. **f** Illustration of two potential LIR motifs in β-dystroglycan using iLIR database. **g** Left: In vitro competition assay: plakoglobin immunoprecipitation from membrane extracts from muscles of fed mice, followed by incubation in vitro with synthetic peptides corresponding to β-dystroglycan’s-LIR domains or scrambled control. β-Dystroglycan LIR-2 peptide efficiently competed with β-dystroglycan on binding to plakoglobin. Right: Densitometric measurement, β-dystroglycan to plakoglobin ratio in protein precipitates following incubation with synthetic peptides. *n* = four independent experiments. *P < 0.05 vs. scrambled. **h** Left: Cross-sections of muscles expressing GFP-β-dystroglycan or GFP-β-dystroglycan-ΔLIR from fasted mice were stained with indicated antibodies. Bar, 20 μm. *n* = three independent experiments. Right: Fluorescence intensity of areas of potential colocalization on the plasma membrane. *n* = 3. *P < 0.05 vs. GFP-β-dystroglycan. Bottom: Colocalization of indicated proteins in region of interest (region of co-occurrence, data are ratio to GFP-β-dystroglycan), and the corresponding Pearson’s correlation coefficients of colocalization. *n* = 7, two independent experiments. *P < 0.05 and **P < 0.005 vs. GFP-β-dystroglycan. **i** Proposed model: Under normal conditions, basal recycling of plakoglobin-DGC-insulin receptor co-assemblies occurs via plakoglobin masking β-dystroglycan LIR domains. During fasting, when autophagy is induced, LC3 competes with plakoglobin on binding to β-dystroglycan’s LIR domains, directing the formed vesicles to the lysosome.

Together, signal transduction through the insulin receptor is initiated at these, DGC-rich, protein clusters on the plasma membrane. Therefore, DGC-insulin receptor coupling must be tightly regulated to prevent inappropriate activation of PI3K-Akt-FoxO signaling and excessive tissue growth, and to efficiently terminate transmission of growth signals from cell surface receptors when cellular energy charge is low. To determine the dynamics of these interactions, we studied mouse muscles during the physiological adaptation to fasting, where blood glucose and insulin levels are low and consequently signaling through the insulin receptor is reduced in all tissues. Immunofluorescence and biochemical analyses showed that the membrane content of plakoglobin, β-dystroglycan, and insulin receptors was reduced during fasting (Fig. 6a, b and Supplementary Fig. 7a). Plakoglobin immunoprecipitation from membrane extracts indicated reduced association with β-dystroglycan during fasting (Fig. 6c, graph), while plakoglobin-insulin receptor association remained intact (Fig. 6c)^21^. Colchicine injection of mice to perturb microtubule polymerization and inhibit autophagy flux (Supplementary Fig. 7b)^45,46^ attenuated this response to fasting, and plakoglobin-insulin receptor-β-dystroglycan-dystrophin co-assemblies accumulated on the muscle membrane (Fig. 6c, d and Supplementary Fig. 7a), indicating that during fasting these proteins are probably lost by the lysosome. Accordingly, the potential colocalization of plakoglobin, insulin receptor, and β-dystroglycan with the lysosomal marker LAMP1 increased during fasting (Supplementary Fig. 7c, d). Interestingly, plakoglobin levels did not increase on the membrane following colchicine injection (Fig. 6d), suggesting that this protein is not degraded but rather accumulates inside the muscle fiber (Fig. 6a), probably bound to autophagic vesicles containing β-dystroglycan and insulin receptor.

### Plakoglobin prevents DGC-insulin receptor loss by autophagy

Previously we have shown that plakoglobin overexpression during fasting leads to a greater activity of the insulin receptor and PI3K-Akt-FoxO signaling^21^, and here we demonstrate that these effects result from stabilization of DGC-insulin receptor assemblies on the plasma membrane (Fig. 6e). To identify the underlying mechanism, we investigated the dynamics of these interactions during fasting. Searching the iLIR database we identified two LC3-interacting region (LIR) motifs in β-dystroglycan, LQFIPV (714–719) and GEYTPL (846–851) (Fig. 6f) that can potentially be recognized by the autophagy marker LC3^47^. By masking β-dystroglycan’s LIR domains, plakoglobin may prevent association of β-dystroglycan with LC3 and the autophagic machinery, consequently preventing β-dystroglycan-insulin receptor loss (Fig. 6e). To test this idea, we established an in vitro competition assay in which we immunoprecipitated plakoglobin from membrane extracts isolated from normal muscles and added in vitro synthetic peptides LIR-1 and LIR-2 (Supplementary Table 3), corresponding to LQFIPV and GEYTPLR LIR motifs in β-dystroglycan, respectively (Fig. 6f, g). After incubation at 4 °C for 1 h, LIR-2 was able to efficiently compete with β-dystroglycan on binding to plakoglobin in vitro, and reduced β-dystroglycan-plakoglobin association by 27% compared with scrambled control peptide (Fig. 6g). These interactions were dynamic because LC3 could be co-precipitated together with plakoglobin and β-dystroglycan from normal muscle, where plakoglobin potentially masks β-dystroglycan’s LIR domains. However, LC3 association with this complex was markedly reduced when LIR-2 peptide was added in vitro (Fig. 6g).

To determine whether β-dystroglycan’s LIR domain is in fact important for association with LC3 and plakoglobin, we generated plasmids encoding GFP-tagged FL-β-dystroglycan or a mutant lacking the LIR motif and investigated the effects of this mutation on colocalization with plakoglobin, insulin receptor, and LC3 during fasting. Following electroporation into muscles, GFP-β-dystroglycan and GFP-β-dystroglycan-ΔLIR showed a similar distribution as did the endogenous protein on the plane of the muscle membrane (Fig. 6h), although GFP-β-dystroglycan-ΔLIR also accumulated inside the muscle fiber probably because the deletion of LIR domain and failure to bind LC3 stabilized this protein. In addition, β-dystroglycan’s LIR domain may be important for association with plakoglobin on the muscle membrane. In fact, the potential colocalization of GFP-β-dystroglycan-ΔLIR with LC3, plakoglobin, and the insulin receptor decreased markedly compared to GFP-tagged FL-β-dystroglycan (Fig. 6h), indicating that β-dystroglycan’s LIR domain is required for stabilization of plakoglobin-DGC-insulin receptor assembly on the muscle membrane (i.e., through association with plakoglobin (Fig. 6g)), and for binding to LC3 when autophagy is induced and plakoglobin-DGC-insulin receptor clusters are lost. It is noteworthy that the overexpressed GFP-β-dystroglycan may colocalize with endogenous insulin receptor and plakoglobin proteins in different proportions, as indicated by the depression of Pearson’s correlation coefficients (PCCs), as often occurs with overexpressed proteins) (Fig. 6h). Thus, under normal conditions, when plakoglobin-DGC-insulin receptor co-assembly is intact, plakoglobin seems to protect β-dystroglycan-insulin receptor co-assembly from LC3-mediated degradation by masking β-dystroglycan LIR domains.

## Discussion

These studies have uncovered protein assemblies of insulin receptors and DGC, whose structural integrity and functional properties are linked and endowed by plakoglobin. We show that plakoglobin stabilizes these structural and signaling modules at costameres, and contributes to maintenance of cell size by promoting tissue integrity through desmin-DGC-laminin axis, and simultaneously regulating signaling through the insulin receptor. Plakoglobin’s stimulation of insulin signaling seems to result from it promoting a tight assembly between the insulin receptor and DGC because decreasing plakoglobin levels in normal muscle reduced DGC-insulin receptor association and insulin signaling, whereas increasing plakoglobin content in diabetic or *mdx* mice muscles enhanced DGC-insulin receptor co-assembly. Consequently, DGC acts as a cellular signaling node containing plakoglobin as a pivotal subunit, and perturbation of plakoglobin function (e.g., by Trim32^21^) probably contributes to the insulin resistance seen in various catabolic states (e.g., untreated diabetes, obesity).

Previous studies demonstrated a correlation between impaired insulin signaling, DGC dissociation, and muscle loss^12,14,15,48^, which can now be explained based on our findings that DGC integrity is linked to insulin receptor activity. For example, reduced sensitivity to insulin is seen in some DMD and Becker muscular dystrophy human patients^7,8,49^, and insulin-related metabolic disorders (e.g., hyperinsulinemia, glucose intolerance, obesity) are seen in muscular dystrophies such as myotonic dystrophy type 1 and 2^49–51^. Similarly, during aging, DGC dissociation significantly contributes to the deterioration in muscle size, strength, and function^52^, which often correlates with the development of insulin resistance^53,54^. In addition, muscle atrophy due to denervation, as occurs during aging, appears to alter muscle sensitivity to insulin^37,55,56^.

Our findings of DGC as a signaling hub are surprising because there had been no prior reports linking the insulin receptor and DGC functionally or structurally. Clustering of signaling receptors in membrane domains is necessary for ligand-dependent signal transmission^57^, and our findings here predict such clustering may occur via association with tissue structural modules. These signaling hubs probably dynamically disassemble and reassemble in response to external cues in a changing environment^57^. Accordingly, we show that stimulation of insulin secretion by the injection of glucose enhances DGC-insulin receptor association and accumulation on the plasma membrane (Fig. 3b, c), whereas reduced signaling through the insulin receptor, as occurs during fasting or diabetes, leads to a fall in the membrane content of these protein assemblies (Figs. 4, 6, and Supplementary Fig. 7a). Plakoglobin stabilizes these heteromultimers on the plasma membrane to maintain tissue integrity because its downregulation (by shJUP) in normal muscle reduced the membrane content of plakoglobin-DGC-insulin receptor co-assemblies and caused atrophy (Fig. 3), and increasing plakoglobin levels in fasting or diabetes stabilized these protein clusters on the muscle membrane and attenuated atrophy (Figs. 5 and 6e). These changes in plakoglobin levels also influence the levels of glycosylated β-dystroglycan (Fig. 3a–c and Supplementary Fig. 4a), whose presence on the plasma membrane is essential for DGC stability^11^.

Here, we report a mechanism that dynamically regulates the engagement of these DGC-rich signaling clusters on the muscle membrane. Under normal conditions, when growth or survival signals are transmitted and insulin/IGF-I-PI3K-Akt pathway is active and inhibits proteolysis by autophagy^2,22,23,58,59^, there is a basal recycling of plakoglobin-DGC-insulin receptor co-assemblies, and plakoglobin prevents their loss by masking β-dystroglycan’s LIR domains. However, during fasting and in disease states, when IGF-I and insulin levels are low, LC3 levels rise and autophagy is induced^23,58,60^, LC3 competes with plakoglobin on binding to β-dystroglycan, and through association with β-dystroglycan’s LIR domains directs the recycling plakoglobin-DGC-insulin receptor-containing vesicles to the lysosome^61^. Consistently, in MKR mouse muscles, impaired insulin receptor activity led to a prominent reduction in the amounts of plakoglobin and DGC components on the membrane (Fig. 4), which probably resulted from accelerated degradation because these proteins did not accumulate in the cytosol and their gene expression was similar in muscles from MKR mice and WT controls (Supplementary Fig. 5a, b). Increasing plakoglobin content (by overexpression) stabilizes DGC-insulin receptor association on the membrane probably by surpassing certain inhibitory effects that prevent engagement of these DGC-rich clusters within membrane domains (e.g., LC3). As a result, the cellular response to insulin is accentuated leading to enhanced protein synthesis and glucose uptake, and reduced protein degradation and atrophy (Fig. 5). Because plakoglobin is expressed in all cells, and since similar interactions were demonstrated in the heart and liver (Supplementary Fig. 2), these functions of plakoglobin are probably general to many tissues.

Therefore, a reciprocal regulation of DGC and the insulin receptor seems to be required to promote DGC stability and insulin receptor activity. β-Dystroglycan probably functions as a scaffold for the insulin receptor as it does for acetylcholine receptor during the formation of neuromuscular junctions in development^62^, and as a signaling hub maintaining insulin receptor activity. In turn, active insulin receptor seems to stabilize the DGC by inhibiting degradation of its constituents via autophagy. Plakoglobin may serve as scaffold protein at the sarcolemma, where the insulin receptor is positioned to interact with the DGC. Here we show that insulin receptors interact with plakoglobin N terminus and β-dystroglycan recognizes adjacent domains, which seem to be masked by plakoglobin C-terminal region^43,63^. Deletion of plakoglobin’s C-terminal domain causes Naxos disease and leads to the formation of large abnormal cadherin-based membrane structures, suggesting that this domain limits the size of membrane structures and regulates protein–protein interactions required for their assembly^43,44^. Consistently, we demonstrate that overexpression of plakoglobin-ΔC mutant enhances association with the DGC and the insulin receptor on the plasma membrane, albeit with no significant effect on insulin receptor activity (Fig. 6). In knock-in mice expressing plakoglobin-ΔC, cardiac function is normal suggesting that this carboxy-terminal domain is dispensable for plakoglobin function in the heart^64^. The effects of this mutation on plakoglobin function and its role in cardiomyopathies merit in-depth study based on the present identification of the protein complexes that plakoglobin forms, and its physiological roles.

In the heart and epithelia, plakoglobin links desmin filaments to desmosomes via desmoplakin^33,34,65^. Skeletal muscle lacks “classic” desmosomes, but contains desmosomal components, which are present on the muscle membrane and can be isolated together with plakoglobin, DGC, and insulin signaling components (Supplementary Table 1)^21^. Unlike β-dystroglycan, plakoglobin is not evenly distributed along the sarcolemma, and instead accumulates in discrete regions by binding to specific receptors to stimulate signaling pathways and to promote cell–ECM contacts as proposed here. These regions are located in close proximity to the nucleus, probably to efficiently translate extracellular signals to transcriptional response. Furthermore, these clusters of plakoglobin-DGC-insulin receptor contain vinculin, which in the skeletal muscle is confined to costameres that link the desmin cytoskeleton and the bound contractile apparatus to laminin at the ECM, hence contributing to muscle mechanical integrity^66^. In fact, the strongest evidence for the importance of plakoglobin to desmin filament stability and thus tissue architecture were our findings that in normal muscle plakoglobin downregulation alone was sufficient to promote desmin depolymerization and atrophy (Fig. 3d–f). Whether loss of intact desmin filaments in skeletal muscle, as occurs in desmin myopathies, affects DGC integrity or signal transmission via the insulin receptor, and whether plakoglobin cellular distribution changes in these diseases are important questions for future research.

Plakoglobin may mediate growth-promoting signals also via the IGF-1-R, although plakoglobin’s beneficial effects on insulin signaling seem to result primarily from it acting on the insulin receptor because in MKR mouse muscles lacking functional IGF-1-R, the accumulation of plakoglobin alone activates insulin signaling (Fig. 5). In any case, plakoglobin seems to enhance insulin potency by triggering intracellular signals, and reduced plakoglobin function is likely to contribute to the development of insulin resistance in type 2 diabetes and perhaps also in DMD^7,8^.

## Methods

### Animals

All animal experiments were consistent with the ethical guidelines of the Israel Council on Animal Experiments and were approved by the Technion Inspection Committee on the Constitution of the Animal Experimentation. Specialized personnel provided animal care in the Institutional Animal facility. We used adult CD-1 male mice (~30 g) (Envigo) in all experiments that did not involve transgenic mice. For colchicine treatment, CD-1 mice were injected intraperitoneally with 0.4 mg/kg colchicine (Sigma C9754) or vehicle at the time of food deprivation, followed by three additional injections every 12 h (total of four injection within 48 h of fasting).

FVB/N male mice (8–10 weeks old) (Envigo) served as control for the MKR diabetic male mice (8–10 weeks old) (kindly provided by Prof. Derek Le Roithe, Rappaport School of Medicine, Technion Institute, Israel). The *mdx* mice muscle were kindly provided by ART BioScience Ltd.

### Antibodies and constructs

The plasmids encoding shRNA against plakoglobin and Lacz were cloned into pcDNA 6.2-GW/EmGFP-miR vector using Invitrogen’s BLOCK-iT RNAi Expression Vector Kit^21^. For generation of GFP-β-dystroglycan-ΔLIR encoding plasmid, a FL mouse β-dystroglycan encoding pEGFP plasmid (kindly provided by Dr. Winder^67^) served as a template for consecutive deletion mutations of β-dystroglycan’s 846–851 and 714–719 residues by PCR using two primer sets: CCTCTGAACCAGGACACTGTGCGGGATGAGGATCCTAACGC together with GCGTTAGGATCCTCATCCCGCACAGTGTCCTGGTTCAGAGG, and CTGGCAGTTGTCGGCACGCACCACCCTCTCCTGGA together with TCCAGGAGAGGGTGGTGCGTGCCGACAACTGCCAG, respectively. The β-dystroglycan-ΔLIR mutant generated by PCR was cloned into pEGFP vector, and the fidelity of the final construct was verified by nucleotide sequencing.

The construct encoding the 6His-plakoglobin was provided by Dr. Yoshitaka Sekido (Nagoya University School of Medicine, Nagoya, Japan). Plakoglobin antibodies were from Genetex (Cat# GTX15153, lot# 821501500, for immunoprecipitation (1:50) and immunoblotting (1:1000)) and Acris (Cat# AP33204SU-N, lot# 610051, for immunofluorescence (1:50)). Anti-dystrophin (Cat# ab15277, lot# GR 256086-2, 1:1000), anti-desmin (Cat# ab8592, 1:1000), anti-vinculin (Cat# ab73412, lot# GR 29133-1, 1:1000), anti-caveolin-1 (Cat# ab2910, lot# GR 235528-4, 1:1000), anti-insulin receptor (Cat# ab5500, lot# GR 242593-6, for immunofluorescence, 1:50), and anti-myosin (Cat# ab7784, 1:1000) were from Abcam. Anti-phospho-insulin receptor (Y1361) (Cat# 3023, lot# 2, 1:1000), anti-insulin receptor (Cat# 3025, lot# 8, for immunoblotting (1:1000) and immunoprecipitation (1:50)), anti-phospho-PI3K (Cat# 4228, lot# 2, 1:1000), anti-phospho-AKT (Cat# 9275, lot# 13, 1:1000), anti-Akt (Cat# 9272, lot# 25, 1:1000), and anti-PI3K (Cat# 4292, lot# 9, 1:1000) were from Cell Signaling Technologies. The β-dystroglycan antibody developed by Glenn E. Morris (RJAH Orthopedic hospital, Oswestry, UK, 1:100) was obtained from the Developmental Studies Hybridoma Bank, created by the NICHD of the NIH and maintained at The University of Iowa, Department of Biology, Iowa city, Iowa (Cat# MANDAG2, clone 7D11, lot# S1ea). Phospho-serine antibody was from ECM Biosciences (Cat# PP2551, lot# 5, 1:1000). Anti-laminin (Cat# L9393, lot# 046m4837v, 1:1000), and anti-GAPDH (Cat# G8795, 1:10,000) were from Sigma. Syntrophin antibody (Cat# Sc-50460, lot# F1109, 1:1000), caveolin-3 antibody (Cat# sc-5310, lot# L1714, 1:1000), MuSK antibody (clone N-19, Cat# sc-6010, 1:1000), and V5 antibody (Cat# R960-2546-0705, lot# 1831141, 1:1000) were from Santa Cruz Biotechnology (Cat# sc-5310, lot# L1714).

### In vivo transfection

For in vivo electroporation approach, 20 μg of plasmid DNA was injected into adult mouse TA muscles, and a mild electric pulse was applied by two electrodes (12 V, 5 pulses, 200 ms intervals)^21^. After 7 days, muscles were dissected and analyzed. In fasting experiments (Fig. 6 and Supplementary Fig. 7), food was removed from cages for 48 h 5 days after muscle electroporation.

For fiber size analysis, cross-sections of muscles were fixed in 4% PFA and fiber membrane was stained with laminin antibody (see details under immunofluorescence). Cross-sectional areas of at least 500 transfected fibers (expressing GFP) and 500 adjacent non-transfected ones in the same muscle section (10 µm) were measured using Metamorph (Molecular Devices) and Imaris (Bitplane) software. Images were collected using a Nikon Ni-U upright fluorescence microscope with Plan Fluor ×20 0.5NA objective lens and a Hamamatsu C8484-03 cooled CCD camera and MetaMorph software.

### Isolation of native plakoglobin complexes from the muscle

To isolate plakoglobin-containing complexes from mouse skeletal muscle, we adapted a protocol by Chorev et al.^26^ with some modifications. Lower limb mouse skeletal muscles (1 g) were homogenized in 19 volumes of cold buffer A (20 mM Tris, pH 7.2, 100 mM KCl, 5 mM EGTA, 40 mM imidazole, 1 mM dithiothreitol (DTT), 1 mM phenylmethylsulfonyl fluoride (PMSF), 3 mM benzamidine, 10 μg/ml leupeptin, 50 mM NaF, 2.7 mM sodium orthovanadate, and 1% Triton X-100). Following centrifugation at 10,000 × *g* for 20 min at 4 °C, the pellet (containing myofibrils, membrane, and cytoskeleton) was washed once in buffer A, and then re-suspended in 10 volumes (v/v) of buffer B (buffer A supplemented with 10 mM ATP) to dissociate myofibrillar myosin from actin thin filaments. After centrifugation at 16,000 × *g* for 10 min at 4 °C, the pellet (mostly containing membrane and cytoskeleton-bound proteins) was washed once in buffer B, re-suspended in 10 volumes (v/v) of buffer C (buffer A at pH 9), and incubated at 37 °C for 30 min with gentle agitation. After centrifugation at 16,000 × *g* for 10 min and 4 °C, the supernatant (containing purified membrane-bound and cytoskeletal proteins) was subjected to 30% ammonium sulfate precipitation, and precipitates were centrifuged at 20,000 × *g* for 30 min at 4 °C. The obtained pellet was kept on ice and the supernatant was subjected to an additional 50% ammonium sulfate precipitation. After a final centrifugation at 20,000 × *g* for 30 min at 4 °C, precipitates from 30% and 50% ammonium sulfate precipitations were re-suspended in 600 µl of buffer A, and 85% of this suspension (containing membrane-cytoskeleton fraction) was loaded onto a Superdex 200 10/300 Gel Filtration column (GE Healthcare Life Sciences, UK) equilibrated with 20 mM Tris, pH 7.2. Proteins were eluted at a flow rate of 0.3 ml/min and 300 μl fractions were collected. Selected plakoglobin-containing fractions (Fig. 1d, fractions #8–11) were combined and loaded onto a 1 ml Resource Q anion exchange column (GE Healthcare Life Sciences, UK), equilibrated with 20 mM Tris, pH 7.2 and 500 mM NaCl. Proteins were eluted by a 0–500 mM NaCl gradient into 0.5 ml fractions, and fractions enriched with plakoglobin (Fig. 1f, lane 7) were analyzed by mass spectrometry, glycerol gradient fractionation, and western blotting.

To identify plakoglobin-binding proteins in whole muscle extracts (Fig. 2a, b), TA muscles from WT mice (200 mg TA from five mice) were electroporated with a plasmid encoding 6His-tagged plakoglobin, and 7 days later, the muscles were dissected, and whole-cell extracts incubated with Ni beads (according to the manufacturer’s instructions) to purify the 6His-tagged plakoglobin and bound proteins. Interacting proteins were then eluted from Ni beads using a histidine gradient (at 50, 80, 100, 150, 200, and 250 mM histidine), and were analyzed by mass spectrometry, sodium dodecyl sulfate-polyacrylamide gel electrophoresis (SDS-PAGE) and immunoblotting.

### Fractionation of muscle, heart, and liver tissues

To obtained whole-cell extracts, lower limb muscles (1 g) were homogenized in lysis buffer (20 mM Tris, pH 7.2, 5 mM EGTA, 100 mM KCl, 1% Triton X-100, 1 mM PMSF, 3 mM benzamidine, 10 μg/ml leupeptin, 50 mM NaF, and 2.7 mM sodium orthovanadate), and following centrifugation at 6000 × *g* for 20 min at 4 °C, the supernatant (containing whole-cell extract) was collected and stored at −80 °C^35^. The pellet was subjected to additional washing steps in homogenization buffer and suspension buffer (20 mM Tris, pH 7.2, 100 mM KCl, 1 mM DTT, and 1 mM PMSF), and after a final centrifugation at 6000 × *g* for 20 min at 4 °C, the pellet (containing purified myofibrils and desmin filaments) was re-suspended in storage buffer (20 mM Tris, pH 7.2, 100 mM KCl, 1 mM DTT, and 20% glycerol) and kept at −80 °C.

To obtain membrane extracts, tissues (muscle, liver, heart) were homogenized in buffer C (20 mM Tris, pH 7.6, 100 mM KCl, 5 mM EDTA, 1 mM DTT, 1 mM sodium orthovanadate, 1 mM PMSF, 3 mM benzamidine, 10 μg/ml leupeptin, and 50 mM NaF) and centrifuged at 2900 × *g* for 20 min at 4 °C. The obtained supernatant (containing membrane and cytosolic proteins) was then centrifuged at 180,000 × *g* for 90 min at 4 °C using a TLA-55 rotor (Beckman Coulter, Brea, CA). The obtained pellet (containing intact membranes) was then re-suspended in buffer M (20 mM Tris-HCl, pH 7.6, 100 mM KCl, 5 mM EDTA, 1 mM DTT, 0.25% sodium deoxycholate, 1% NP-40, 1 mM sodium orthovanadate, 10 μg/ml leupeptin, 3 mM benzamidine, and 1 mM PMSF), and incubated at 4 °C for 20 min with agitation (220 × *g*) to solubilize membrane proteins. After a final centrifugation at 100,000 × *g* for 30 min at 4 °C, the supernatant (i.e., purified membrane fraction) was collected and stored at −80 °C.

### Glycerol gradient fractionation

Equal amounts of muscle homogenates were layered on a linear 10–40% glycerol gradient containing 20 mM Tris, pH 7.6, 5 mM EDTA, pH 7.4, 100 mM KCl, 1 mM DTT, 0.24% sodium deoxycholate, and 1 mM sodium orthovanadate. Following centrifugation at 131,300 × *g* for 24 h at 4 °C using a MLS-50 Swinging-Bucket rotor (Beckman Coulter, Brea, CA) to sediment protein complexes, 300 μl fractions were collected, and alternate fractions were subjected to trichloroacetic acid precipitation (10%) for 24 h at 4 °C. Precipitates were then analyzed by SDS-PAGE and immunoblotting.

### Protein analysis

For immunoblotting and immunoprecipitation, tissue homogenates and fractions of isolated plakoglobin-containing complexes were resolved by SDS-PAGE and immunoblotting with specific antibodies^21^. Immunoprecipitation experiments using specific antibodies (control sample contained mouse IgG) were performed overnight at 4 °C, and protein A/G agarose was then added for 4 h. To remove nonspecific or weakly associated proteins, the resulting precipitates were washed extensively with 10 bed volumes of each of the following buffers: buffer A (50 mM Tris-HCl, pH 8, 500 mM NaCl, 0.1% SDS, 0.1% Triton, 5 mM EDTA), buffer B (50 mM Tris-HCl, pH 8, 150 mM NaCl, 0.1% SDS, 0.1% Triton, 5 mM EDTA), and buffer C (50 mM Tris-HCl, pH 8, 0.1% Triton, 5 mM EDTA). Protein precipitates were eluted in protein loading buffer containing 50 mM DTT and were analyzed by immunoblotting.

### Immunofluorescence labeling of frozen muscle sections

Frozen cross-sections of mouse TA were cut at 10 or 30 μm (for Z-stack and 3D modeling), fixed in 4% paraformaldehyde (PFA), and incubated in blocking solution (0.2% bovine serum albumin and 5% normal goat serum in PBS-T) for 1 h at room temperature (RT). Immunofluorescence was performed using plakoglobin (1:50), laminin (1:50), vinculin (1:50), β-dystroglycan (1:30), or insulin receptor (1:30) antibodies overnight at 4 °C, followed by 1 h incubation at RT with secondary antibodies conjugated to Alexa Fluor 568, 647 or DyLight 488 (1:1000). Both primary and secondary antibodies were diluted in blocking solution. Confocal images were collected and analyzed using an inverted LSM 710 laser scanning confocal microscope (Zeiss, Oberkochen, Germany) with a Plan-Apochromat ×63 1.4 NA objective lens and BP 593/46, BP 525/50, and 640–797 filters, and Imaris 8.2 software. STED images were collected using a Leica DMi8 CS Bino super-resolution microscope (TCS SP8 STED) with a Plan-Apochromat ×100 1.4 NA oil objective lens, WLL (white light laser, covers the spectral range from 470 to 670 nm) and the Most Versatile Beamsplitter AOBS, and Leica 3 HyD SP GaAsP-detector.

PLA assay was performed in humid chamber according to the manufacturer’s instructions (Duolink in situ Red, through Sigma). Briefly, muscle cross-sections were incubated with β-dystroglycan and insulin receptor antibodies overnight at 4 °C, and the control sample was incubated with β-dystroglycan antibody alone. Following incubation with probe-linked secondary antibodies (1 h at 37 °C), the hybridized complementary probes (those that are within 20–100 nm) were ligated (30 min at 37 °C) to form circular DNA. Finally, rolling circle amplification reaction and incorporation of red fluorescently labeled nucleotides were allowed to proceed for 3 h at 37 °C. Red fluorescent spots were visualized and images collected using an inverted LSM 710 laser scanning confocal microscope (Zeiss, Oberkochen, Germany) with a Plan-Apochromat ×63 1.4 NA objective lens and BP 593/46 filter, and Imaris 8.2 software.

### [^3^H]-2-deoxyglucose uptake by the skeletal muscle

For glucose uptake assay, we used a modified protocol adapted from Fernandez et al.^4^, Lauro et al.^68^, and Witczak et al.^69^. Mice were fasted for 12 h and euthanized with CO_2_. TA muscles were carefully removed, weighted, and incubated for 2 h in Krebs-Ringer bicarbonate (KRB) buffer (117 mM NaCl, 4.7 mM KCl, 2.5 mM CaCl_2_, 1.2 mM MgSO_4_, 1.2 mM KH_2_PO_4_, and 25 mM NaHCO_3_), which was pre-equiliberated to 95% O_2_ and 5% CO_2_ and adjusted to 37 °C. To measure glucose uptake, dissected muscles were transferred to KRB buffer containing 1.5 μCi/ml of [^3^H]-2-deoxyglucose, 200 nM insulin (Sigma), 0.1 mM 2-deoxy-d-glucose (Sigma), and 7 mM manitol (J.T. Baker) for 45 min at 95% O_2_, 5% CO_2_, and 37 °C. Cytochalasin B (20 nM), a glucose transport inhibitor (Sigma), was added to control tubes. Then, muscles were quickly blotted on a filter paper and frozen in liquid nitrogen to stop the reaction. To measure glucose uptake, muscles were homogenized in 500 μl lysis buffer (20 mM Tris, pH 7.4, 5 mM EDTA, 10 mM sodium pyrophosphate, 100 mM NaF, 2 mM sodium orthovanadate, 10 μg/ml aprotinin, 10 μg/ml leupeptin, 3 mM benzamidine, and 1 mM PMSF) and rates of [^3^H]-2-deoxyglucose uptake were assessed in muscle homogenates using scintillation counter^69^.

For glucose bolus experiments, d-glucose (1 mg/g body weight) was administered to mice by retro-orbital injection. TA muscles were collected 25 min after injection and kept frozen for further analysis.

### Mass spectrometry analysis

To identify the components comprising plakoglobin-containing multiprotein assemblies, fractions eluted as a high-molecular-mass peak from the gel filtration column (Fig. 1c), as a sharp peak from the anion exchange column (Fig. 1f lane 7), or as 6His-tagged plakoglobin-bound proteins (Fig. 2a) were analyzed by SDS-PAGE and Coomassie Blue staining. The stained protein bands were reduced with 3 mM DTT at 60 °C for 30 min, modified with 10 mM iodoacetamide in 100 mM ammonium bicarbonate in the dark at RT for 30 min, and then digested in 10% acetonitrile and 10 mM ammonium bicabonate with modified trypsin (Promega) at a 1:10 enzyme-to-substrate ratio overnight at 37 °C. The tryptic peptides were desalted using C18 tips (homemade stage tips), dried, and re-suspended in 0.1% formic acid. The peptides were resolved by reverse-phase chromatography on 0.075 × 180-mm fused silica capillaries (J&W) and packed with Reprosil reversed phase material (Dr. Maisch GmbH, Germany). The peptides were eluted with linear 60 min gradient of 5–28%, 15 min gradient of 28–95%, and 15 min at 95% acetonitrile with 0.1% formic acid in water at flow rates of 0.15 μl/min. Mass spectrometry was performed by Q Exactive plus mass spectrometer (Thermo) in a positive mode using repetitively full MS scan, followed by collision induces dissociation (HCD) of the 10 most dominant ions selected from the first MS scan. The mass spectrometry data was analyzed using the Proteome Discoverer 1.4 software with Sequest (Thermo), and Mascot (Matrix Science) algorithms against mouse Uniprot database. Results were filtered with 1% false discovery rate. Semiquantitation was done by calculating the peak area of each peptide based on its extracted ion currents, and the area of the protein is the average of the three most intense peptides from each protein.

### Quantitative real-time PCR

Total RNA was isolated from muscle using TRI reagent (Sigma, #T9424) and served as a template for synthesis of cDNA (complementary DNA) by reverse transcription (Quanta Script cDNA Synthesis Kit, #84005, #84002). Real-time quantitative PCR was performed on mouse target genes using specific primers (Supplementary Table S4) and PerfecTa SYBR Green FastMix (Quanta 84071) according to the manufacturer’s protocol.

### Statistical analysis and image acquisition

Data are presented as means and error bars indicate SEM. The statistical significance was accessed by one-tailed Student’s *t* test. Images were processed by Adobe Photoshop CS5, version 12.1. Quantity One algorithm (Bio-Rad Laboratories, version 29.0) was used for densitometric measurements of protein bands’ intensity.

Confocal images were collected and analyzed using an inverted LSM 710 laser scanning confocal microscope (Zeiss, Oberkochen, Germany) with a Plan-Apochromat ×63 1.4 NA objective lens and BP 593/46, BP 525/50, and 640–797 filters, and Imaris 8.2 software. Colocalization (PCC) and percent colocalization in region of interest (is the region of cooccurrence) were quantified between signal intensities of β-dystroglycan and plakoglobin, β-dystroglycan and insulin receptor, and plakoglobin and insulin receptor using confocal images, and image analysis was performed with Imaris (Bitplane, ver 9.1.2) by ImarisColoc module. This pixel-based module provided a cut-off threshold for separating signal from background pixels and defined the overlap between any two-color channels (colocalized pixels) of the image. Because PCC values did not reach 1, we conclude that these proteins may colocalize on the plasma membrane.

STED images were collected using a Leica DMi8 CS Bino super-resolution microscope (TCS SP8 STED) with a Plan-Apochromat ×100 1.4 NA oil objective lens, WLL (covers the spectral range from 470 to 670 nm) and the Most Versatile Beamsplitter AOBS, and Leica 3 HyD SP GaAsP-Detector. STED images were analyzed using the STED image reconstruction module of Leica. Sub-diffraction spots cooccurrence analysis and visualizations was implemented using the Imaris 9.1.2 software (Oxford, Bitplane). IMARIS spots module was set to identify sub-diffraction spots as particles with 150 nm diameter and quality threshold value above 3000; to identify triple spots cooccurrence, the insulin receptor staining was used as baseline to filter-in the other proteins beyond intensity threshold value of 1500, and to refine the positioning of the triple particles. Moreover, a built-in Imaris plugin spot cooccurrence algorithm (requiring ImarisXT module) was used to identify triple spots centers with maximum distance of 250 nm.

## Supplementary information

Supplementary Information

## Data Availability

The datasets generated and analyzed during the current study, as well as materials and associated protocols, are available from the corresponding author on request. The source data underlying Figs. 1b, d, f, 2b–g, 3a–g, and 4a–j, and Supplementary Figs. 1a, b, 2a–d, 4a, 5a–b, d–e, 6b–d, 7a–d are provided as a Source Data file. Accession code availability: the mass spectrometry proteomics data from isolation of plakoglobin-containing protein complexes have been deposited to the ProteomeXchange Consortium via the PRIDE partner repository^28^ with the database identifier PXD016989.
