# Supplementary material for: A signaling hub of insulin receptor, dystrophin glycoprotein complex and plakoglobin regulates muscle size
Source: Nat Commun. 2020 Mar 13;11:1381. doi: 10.1038/s41467-020-14895-9 (PMC7070008; doi:10.1038/s41467-020-14895-9)
Supplement: Supplementary file 1 — Supplementary Information [file 41467_2020_14895_MOESM1_ESM.pdf]

A signaling hub of insulin receptor dystrophin glycoprotein complex and plakoglobin regulates muscle size

Yara Eid Mutlak[†], Dina Aweida[†], Alexandra Volodin, Bar Ayalon, Nitsan Dahan, Anna Parnis, and Shenhav Cohen[*].

Faculty of Biology, Technion Institute of Technology, Haifa, Israel

[†] These authors contributed equally

[*] Correspondence to Dr. Shenhav Cohen:

Faculty of Biology, Technion Institute of Technology

Haifa 32000, Israel

Tel: 972-4-8294214

shenhavc@technion.ac.il

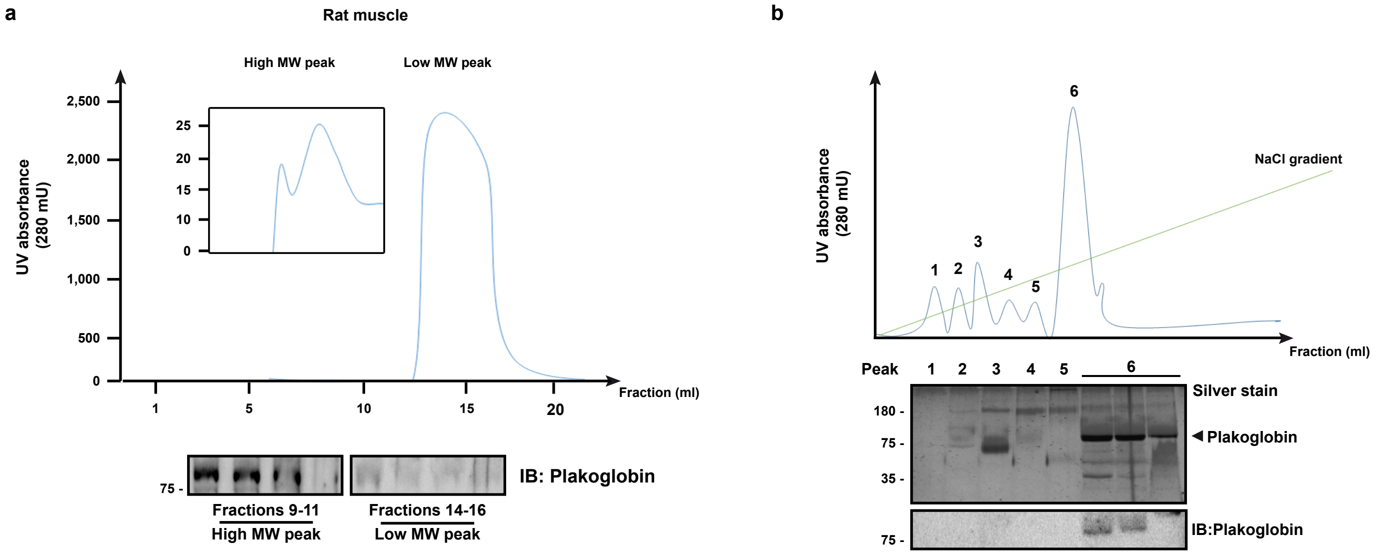

Supplementary Figure 1. Isolation of plakoglobin-containing protein complexes from rat skeletal muscle.

(**a**) **Top:** size-exclusion chromatography of membrane-cytoskeleton fraction from rat lower limb muscles yielded two distinct protein peaks of high and low MW similar to the ones obtained from analysis of mouse skeletal muscle. **Bottom:** fractions from the high (fractions 9-11) and low (fractions 14-16) MW protein peaks were analyzed by SDS-PAGE and immunoblotting with plakoglobin antibody. Black lines indicate the removal of intervening lanes for clarity and presentation purposes.

(**b**) **Top:** fractionation 9-11 in (**a**) were loaded on a resource Q column, and protein complexes were isolated by a 0-500mM NaCl gradient. **Bottom:** analysis of eluted peak fractions by SDS-PAGE and silver staining or immunoblotting using plakoglobin antibody.

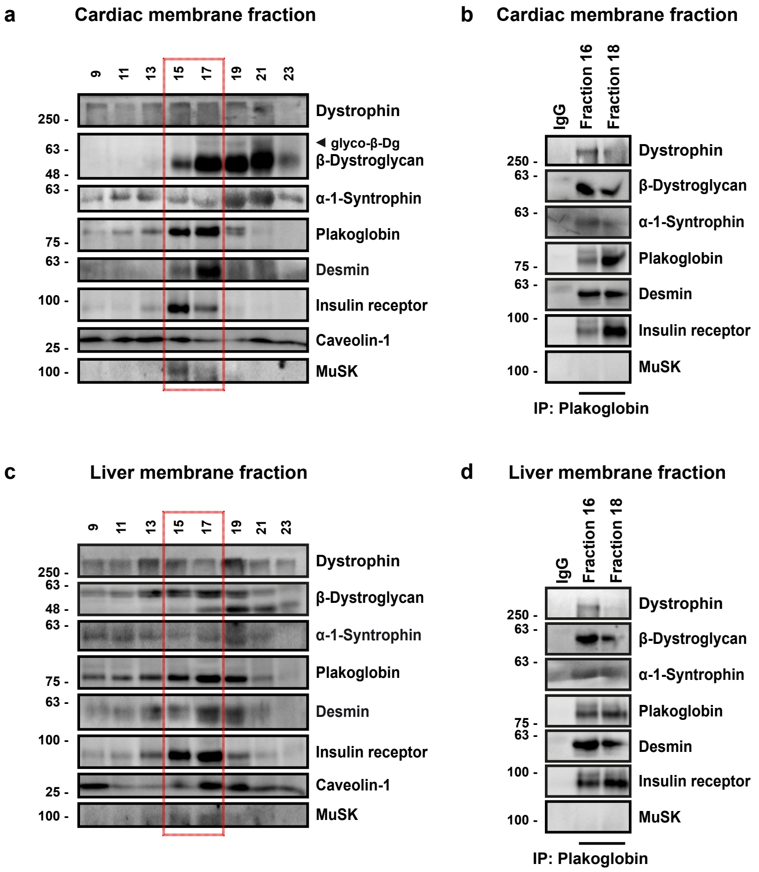

Supplementary Figure 2. Plakoglobin binds DGC components and the insulin receptor in heart and liver.

Isolated membrane fraction from heart (**a**) and liver (**c**) were analyzed by glycerol gradient fractionation and immunoblotting. Plakoglobin, DGC components, the insulin receptor, caveolin-1, desmin, and MuSK sediment to the same glycerol gradient fractions (marked by a red rectangle). n=two independent experiments.

(**b**) Proteins co-purified with anti-plakoglobin from glycerol gradient fractions #16 and #18 illustrated in (**a**) were detected by immunoblotting.

(**d**) Proteins co-purified with anti-plakoglobin from glycerol gradient fractions #16 and #18 illustrated in (**c**) were detected by immunoblotting.

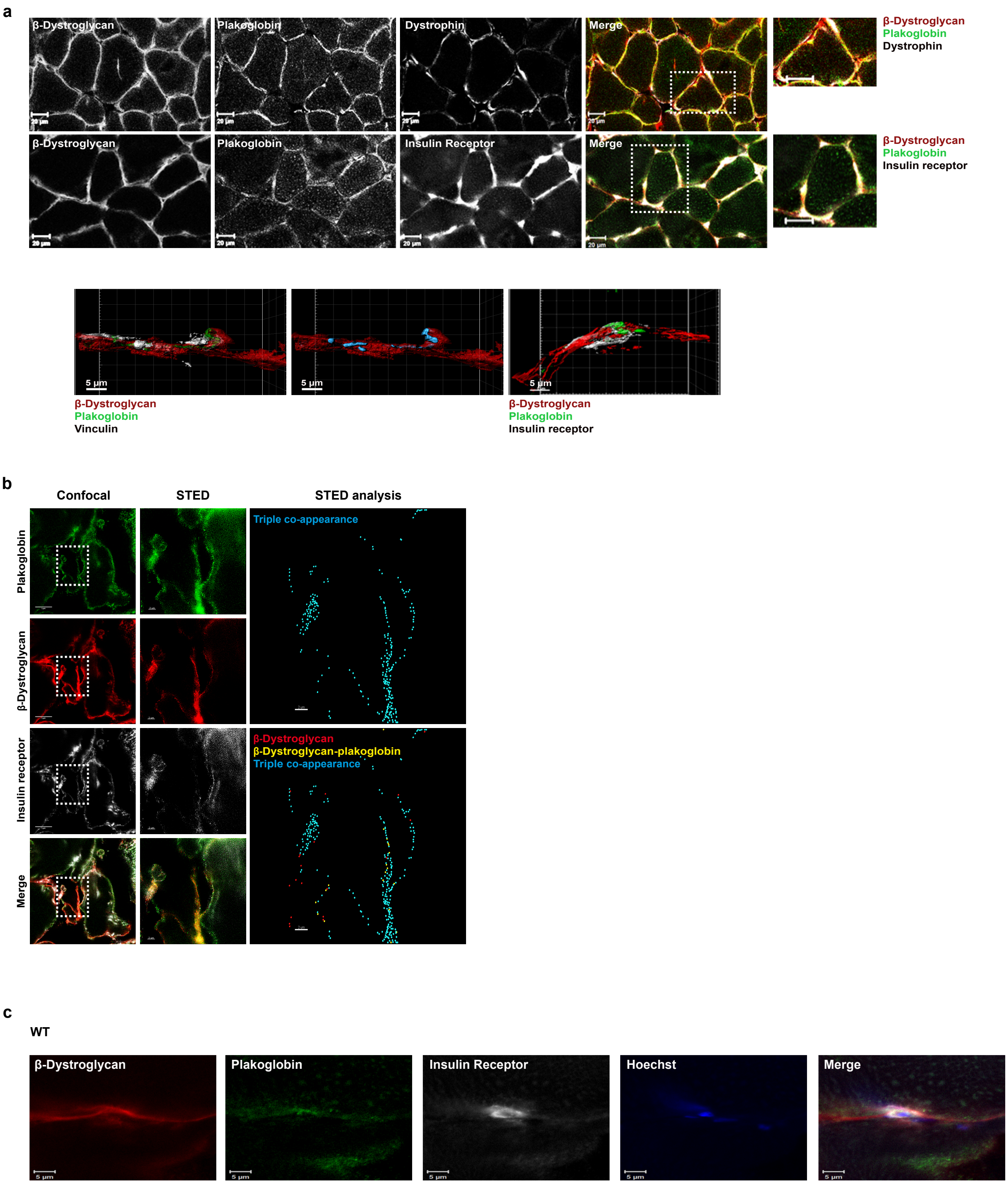

Supplementary Figure 3. Plakoglobin-insulin receptor-DGC co-assembly is localized at sarcomeres in close proximity to the nucleus.

(**a**) Plakoglobin, β-dystroglycan, dystrophin, vinculin, and the insulin receptor co-appear at costameres on skeletal muscle membrane. **Top:** representative confocal images of TA muscle cross sections using the indicated antibodies. Bar, 20 µm. n= three independent experiments indicating co-appearance of these proteins. **Bottom:** 3D modeling using Imaris software of the areas where β-Dystroglycan and plakoglobin co-appear with Vinculin or the insulin Receptor. In blue are areas of triple-co-appearance. Bar, 5µm.

(**b**) Confocal and STED images of TA muscle cross sections stained with the indicated antibodies. Bars, 5µm (Confocal) and 2µm (STED). STED analysis is an annotated image in which all three proteins are detected using the spots module of Imaris software (white, red and green spheres). The double and triple co-occurrence spots are presented in yellow and blue, correspondingly.

(**c**) Cross sections of normal TA muscles were stained with antibodies against plakoglobin, β-dystroglycan and insulin receptor. Bar, 5µm. n=three independent experiments (representative confocal images are shown).

a

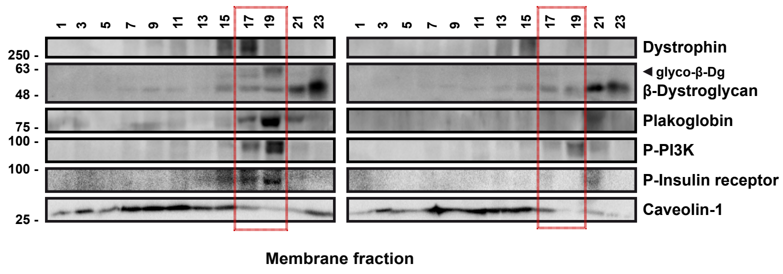

b

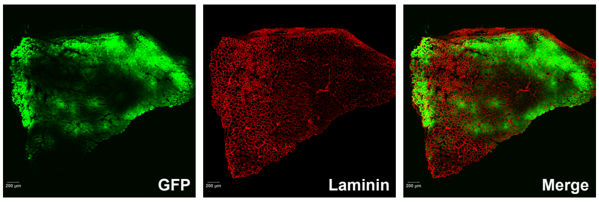

Supplementary Figure 4. Plakoglobin knockdown with shJUP promotes DGC-insulin receptor dissociation.

(**a**) Purified membranes from TA muscles expressing shLacz or shJUP (contralateral limbs) from mouse injected with saline were analyzed by glycerol gradients and immunoblot. Red rectangle marks fractions of plakoglobin-containing complex with glycosylated-β-dystroglycan. n=three independent experiments.

(**b**) Representative images for a muscle transfected with shJUP to evaluate transfection efficiency. For our biochemical studies we use muscles that are at least 60-70% transfected.

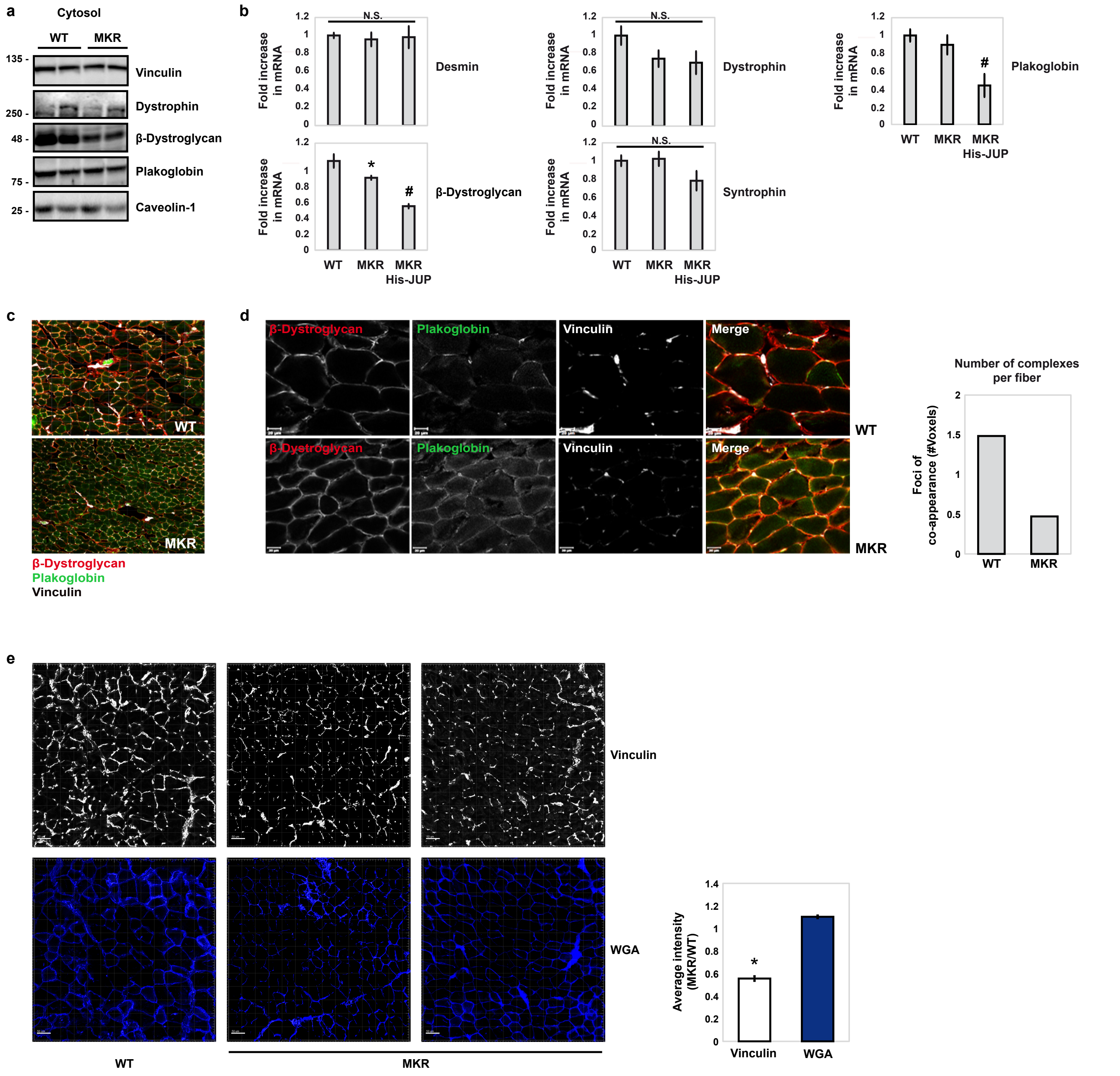

Supplementary Figure 5. In MKR mice muscles, the levels of plakoglobin on the membrane are lower than WT.

(**a**) Soluble fractions of MKR and WT mice muscles were analyzed by SDS-PAGE and immunoblot analysis using antibodies against the indicated proteins. n= two independent experiments (a representative blot is shown). Each lane represents one muscle from one mouse.

(**b**) RT-PCR of mRNA preparations from WT and MKR muscles expressing 6His-plakoglobin or control using primers for desmin, β-dystroglycan, dystrophin, syntrophin, and plakoglobin. Data are plotted as the mean fold change relative to control ±SEM. n=4.

(**c**,**d**) Cross sections of WT and MKR mice TA muscles were stained with plakoglobin, β-dystroglycan and vinculin antibodies. Bar, 100μm (**c**) and 20μm (**d**) Areas of co-appearance were quantified using Imaris software based on fluorescent intensity of a cross section of a whole muscle (tile images, 10X magnification) (**d**, right). n=295 fibers for WT, n=542 fibers for MKR.

(**e**) Cross sections of WT and MKR mice TA were stained with vinculin antibody and WGA. Bar, 50μm. Right: fluorescent intensity was quantified using Imaris software, and data from three independent experiments is depicted in a graph. Mean intensity is presented ±SEM. n=3

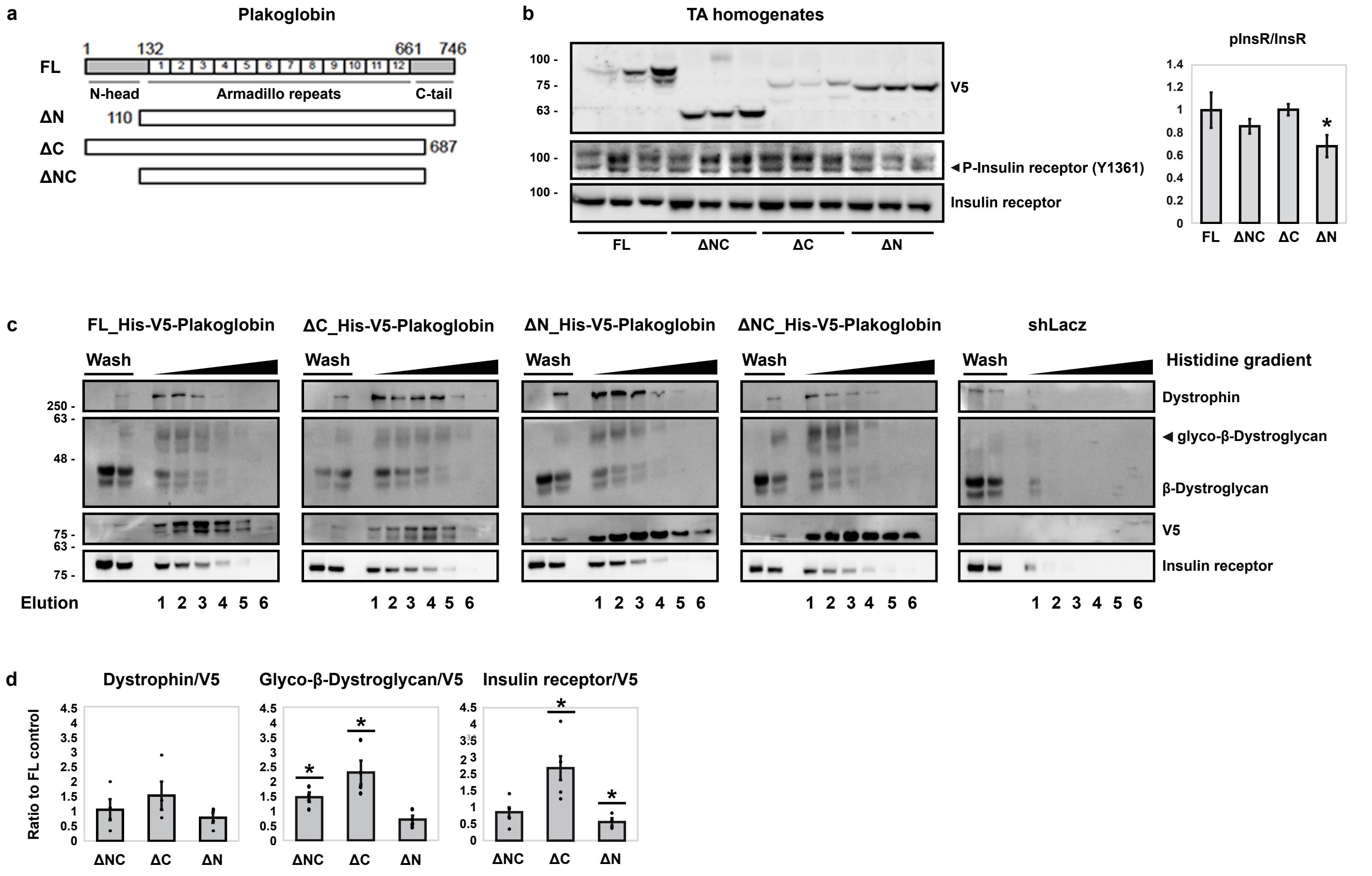

Supplementary Figure 6. Identification of interaction sites between plakoglobin, β-dystroglycan and the insulin receptor.

(**a**) Illustration of plakoglobin truncation mutants.

(**b,c,d**) Insulin receptor binds to plakoglobin N terminus, and adjacent regions in plakoglobin are recognized by □-dystroglycan. TA muscles from fed mice were electroporated with plasmids encoding 6His-V5-plakoglobin (FL), or its truncation mutants lacking the C-terminus (ΔC), the N-terminus (ΔN) or both (ΔNC).

(**b**) Loss of plakoglobin N-terminus reduces insulin receptor activity. Left: soluble extracts from transfected muscles were analyzed by immunoblotting using specific antibodies. Each lane represents one muscle from one mouse. n=two independent experiments (a representative blot is shown). **Right:** densitometric measurement of presented blots. Data are mean ±SEM, n=3. * $p<0.05$. vs. FL, by one-tailed t-test.

(**c**) Isolated membrane fractions from transfected TA muscles were loaded on Nickel column in parallel, and bound proteins were purified with histidine gradient. Lanes 1-6 represent elution fractions # 1-6 with increasing histidine concentration (20-250mM). Data is compared to control muscle expressing shLacz. n=five independent experiments (representative blots are shown).

(**d**) Densitometric measurement of insulin receptor, glycosylated-β-dystroglycan, and dystrophin ratios to V5-tagged plakoglobin mutants in the Nickel column eluates. Ratios to V5-tagged FL-plakoglobin are presented. Data are mean ±SEM, n=5. * $p<0.05$. vs. FL, by one-tailed t-test.

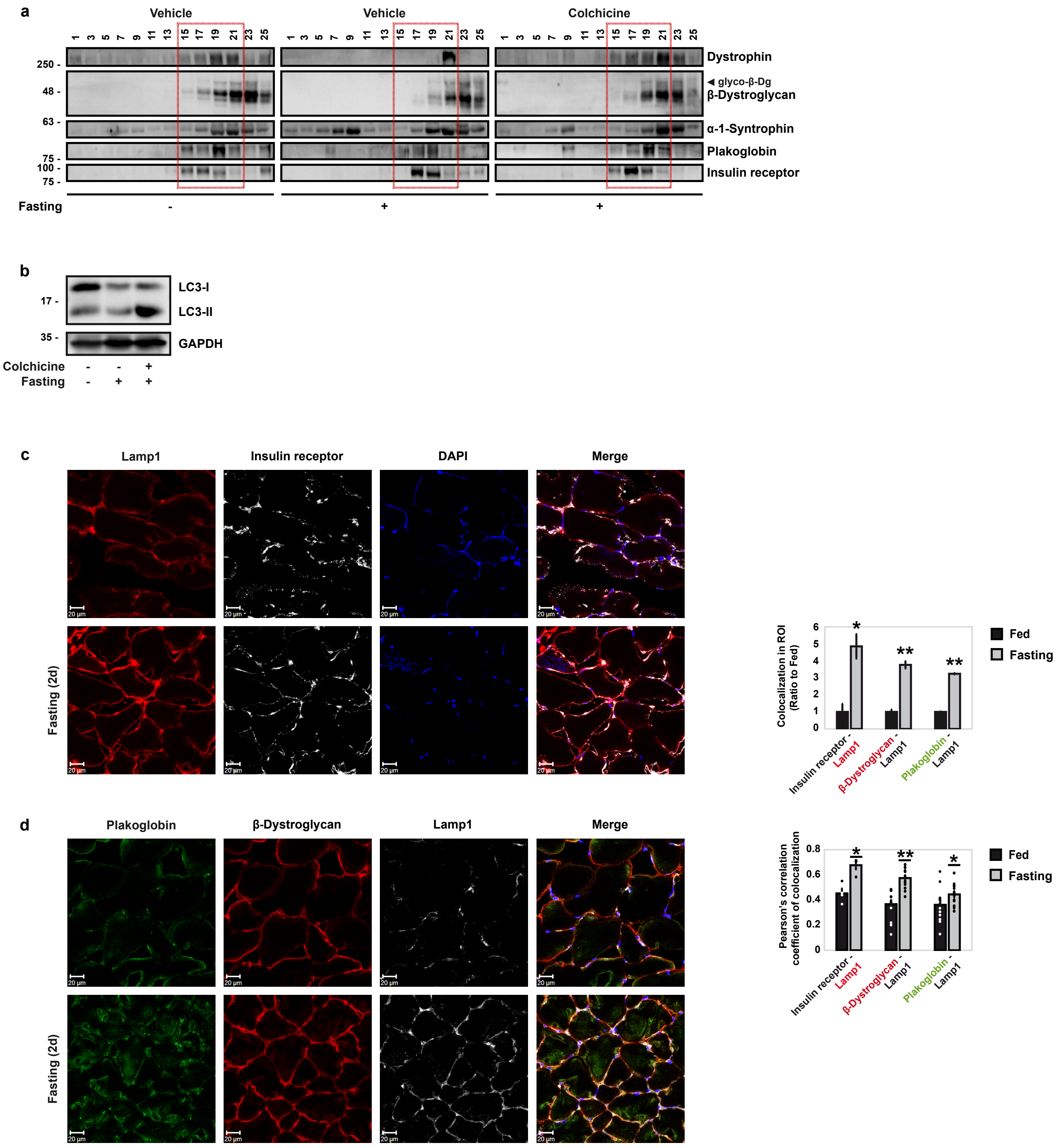

Supplementary Figure 7: Plakoglobin, DGC, and insulin receptor co-appear with autophagy markers during fasting.

(**a**) Membrane extracts from TA muscles from fed or fasted (2 d) mice injected with colchicine or vehicle were analyzed by glycerol gradient, SDS-PAGE and immunoblot. n=four independent experiments.

(**b**) Whole muscles homogenates from fed or fasted (2 d) mice injected with colchicine or vehicle were analyzed by SDS-PAGE and immunoblot. n=two independent experiments.

(**c-d**) **Left:** Cross sections of mouse TA muscles from fed and fasted (2 d) mice were stained with plakoglobin, β-dystroglycan, insulin receptor, and Lamp1 antibodies. Bar, 20μm. n=three independent experiments (representative confocal images are shown). **Right:** co-appearance of indicated proteins in Region of Interest (ROI, is the region of coappearance, data is presented as ratio to Fed), and the corresponding Pearson's correlation coefficients of colocalization. n=5 for insulin receptor-Lamp1 and n=13 for plakoglobin-Lamp1 and β-dystroglycan-Lamp1. Two independent experiments. * $P<0.05$ and ** $P<0.005$ vs. Fed, by one-tailed t-test. Data are represented as mean ±SEM.

SUPPLEMENTARY TABLES

Supplementary Table 1. Membrane, cytoskeletal, and myofibrillar components that were co-purified with plakoglobin from skeletal muscle.

Protein name	Gene	# Unique peptides
Plakoglobin	JUP	15
Sarcoglycan, delta	SGCD	3
Laminin, beta 2	LAMB2	4
Laminin, gamma 1	LAMC1	17
Laminin Subunit Beta 1	LAMB1	21
Spectrin alpha 2	SPNA2	7
Spectrin beta 1	SPTBN1	8
Desmin	DES	9
Plectin	PLEC	36
Actinin, alpha 1	ACTN1	23
Actinin alpha 2	ACTN2	29
Actinin alpha 3	ACTN3	77
Actinin alpha 4	ACTN4	12
Plakophilin 1	PKP1	2
Desmoglein 1	DSG1C	2
Desmoplakin	DSP	25
Cadherin 13	CDH13	2

Native protein assemblies of plakoglobin and bound proteins were isolated from membrane-cytoskeletal preparations of lower limb mouse muscles by size exclusion chromatography and anion-exchange column. Components that were co-purified with plakoglobin as a major protein peak were identified by mass spectrometry.

Supplementary Table 2. Plakoglobin binds membrane, cytoskeletal, myofibrillar and insulin signaling components in skeletal muscle homogenates (6,000g supernatant).

Protein name	Gene (NCBI)	# Unique peptides	Present in high MW peak
Plakoglobin	JUP	16	+
Dystrophin	DMD	20	
Sarcoglycan, alpha	SGCA	3	
Sarcoglycan, beta	SGCB	2	
Sarcoglycan, delta	SGCD	2	+
Alpha-1-syntrophin	SNTA1	9	
Beta-2-syntrophin	SNTB2	4	
Dystroglycan	DAG1	3	
Nitric oxide synthase 1	NOS1	3	
Vinculin	VCL	6	
Caveolin 1	CAV1	5	
Caveolin 3	CAV3	2	
Laminin, beta 2	LAMB2	3	+
Laminin, gamma 1	LAMC1	2	+
Spectrin alpha 2	SPNA2	26	+
Spectrin beta 1	SPNB1	13	+
Insulin receptor substrate 1	IRS1	7	
Insulin-like growth factor 2 receptor	IGF2R	9	
Insulin-like growth factor binding protein	IGFALS	6	
PI3K Catalytic Subunit Type 3	PIK3C3	1	
PI3K Catalytic Subunit Gamma	PIK3CG	2	
PI3K-p85	PIK3R1	4	
Desmin	DES	26	+
Plectin	PLEC	147	+
Actinin, alpha 1	ACTN1	6	+
Actinin alpha 2	ACTN2	1	+
Actinin alpha 3	ACTN3	2	+
Actinin alpha 4	ACTN4	12	+
Cadherin 13	CDH13	6	+

Affinity purification of 6His-tagged plakoglobin and associated proteins from the soluble fraction of mouse lower limb muscles. Mass spectrometry analysis identified membrane, cytoskeletal and myofibrillar components (also present in the high MW peak, see Table I), as well as component of PI3K-Akt signaling.

Supplementary Table 3. Synthetic peptides corresponding to β-dystroglycan's LIR domains that were used in the present study.

**Name**	**LIR motif**	**Pattern**	**Position (residues, mouse)**	**Synthetic peptide**
LIR-1	WxxL	LQFIPV	714-719	CRH**LQFIPV**
LIR-2	xLIR	GEYTPL	846-851	DTM**GEYTPL**R
Scrambled				LRVQCFHP

Supplementary Table 4. qPCR primers used in the present study.

**DNA**	**Gene**	**Sequence**
PCR primer Forward	Desmin	GGATGGAGAGGTTGTCAGCG
PCR primer Reverse	Desmin	GTGGCTGGGTGTGATATCCG
PCR primer Forward	β-Dystroglycan	GTGAACTGGCTGCTGGATACT
PCR primer Reverse	β-Dystroglycan	ATGATGTGGTCCCAGGGTTG
PCR primer Forward	Dystrophin	AAGAGGAAGAAATGCCCCCG
PCR primer Reverse	Dystrophin	CCATGCGGGAATCAGGAGTT
PCR primer Forward	Syntrophin	TGATGGCACGAGTCTCCTTT
PCR primer Reverse	Syntrophin	GGCCACAAGATGAACGATCC
PCR primer Forward	Plakoglobin	CTGTGTGCCCTCTGTAAGCA
PCR primer Reverse	Plakoglobin	GAACTGTCCTCGCCTGAGAC
PCR primer Forward	RPLPO	GCGACCTGGAAGTCCAACTA
PCR primer Reverse	RPLPO	ATCTGCTTGGAGCCCACAT
